# 20-MHz high-intensity focused ultrasound for tattoo removal without pigment-specific optical targeting and with evidence for an ablation-associated transepidermal extrusion pathway

**DOI:** 10.3389/fmed.2026.1869606

**Published:** 2026-09-28

**Authors:** Ju-Eun Hong, Seung Phil Hong, Ki-Ju Kwon, So-Min Kim, Somin Lee, Saerom Sung, Hyunmin Yang, Sang-Hyeon Yoo, Ha-Neul Jang, Ki-Jong Rhee, Inyong Kwon, Chul Hee Min, Sangwoon Jeong, Misun Kim, Hyejung Cha, Sunghyun Kim, Young Min Park, Mansoon Yoon, Hyun Joon Choi, Sei Hwan You

**Affiliations:** 1 Medical Device Business Division, ECO DM LAB Co., Ltd., Cheongju, Republic of Korea; 2 Department of Biomedical Laboratory Science, College of Health Sciences, Yonsei University, Wonju, Republic of Korea; 3 Department of Dermatology, Wonju Severance Christian Hospital, Yonsei University Wonju College of Medicine, Wonju, Republic of Korea; 4 Department of Anatomy, Konkuk University School of Medicine, Glocal Campus, Chungju-si, Republic of Korea; 5 Department of Radiation Convergence Engineering, Yonsei University, Wonju, Republic of Korea; 6 Department of Radiation Oncology, Wonju Severance Christian Hospital, Yonsei University Wonju College of Medicine, Wonju, Republic of Korea

**Keywords:** high-intensity focused ultrasound, non-optical tattoo treatment, radiotherapy tattoo, skin wound healing, tattoo pigment reduction, tattoo removal, transepidermal extrusion

## Abstract

**Introduction:**

Radiotherapy tattoos can impose a cosmetic burden, while laser removal requires selection of optical wavelengths according to the absorption characteristics of individual tattoo pigments. We evaluated a handheld 20 MHz high-intensity focused ultrasound (HIFU) device as a potential non-optical approach to reducing red, black, and blue tattoo pigments.

**Methods:**

Thermal injury zone (TIZ) geometry was characterized *ex vivo* in avian muscle and full-thickness porcine skin tissue. Rat dot tattoos (red/black/blue) received a single-session exposure (10 shots; 6 W, 1,000 ms) and were followed for 21 days with histology and morphometry. In Yucatan minipigs, multispectral 3D imaging quantified acute (minutes) and chronic (Day 4–98) responses in normal and tattooed skin, and was extended to multicolor tattoos with >100-day follow-up. A pilot substudy applied a hydrocolloid dressing.

**Results:**

*Ex vivo* TIZs scaled with energy, with restricted axial expansion in porcine tissue. In rats, frosting progressed to crust formation (Days 7–14) and pigment lightening by Day 21; pigment debris was observed near the dermo-epidermal junction and within crusts, consistent with transepidermal elimination. TIZ depth decreased from 730 ± 196 *μ*m at 1 h to 362 ± 125 *μ*m at Day 21, while peripheral ring-like traces were noted in some sites. In minipigs, tattoo contrast decreased (ΔE^*^_ab_ 9.87 → 3.30 by Day 98), and similar contrast reductions were observed across red, black, and blue tattoos by Day 101–105. Hydrocolloid coverage was associated with earlier eschar detachment and faster normalization by Day 14.

**Discussion:**

These feasibility data suggest that 20 MHz HIFU may support an ablation-associated transepidermal extrusion pathway for reducing red, black, and blue tattoo pigments without requiring pigment-specific optical wavelength matching. The findings provide preliminary histology-linked and imaging-supported evidence for this non-optical tattoo-treatment approach, while underscoring the need for larger controlled studies and dose optimization to improve cosmetic outcomes and minimize residual traces.

## 1 Introduction

Permanent skin tattoos have been widely used in radiotherapy as external landmarks to support reproducible patient positioning and radiation field alignment throughout a treatment course (1, 2). Such marks are typically placed prior to treatment to facilitate consistent setup relative to simulation images and patient anatomy (1). However, once radiotherapy is completed, visible tattoos may be perceived as undesirable by some patients and could contribute to psychosocial burden, particularly when they function as a persistent visual reminder of prior illness (2, 3). Dermatologic sequelae of cancer therapies, including permanent cutaneous marks and disfigurement, have been associated with anxiety, depressive symptoms, and reduced quality of life in survivorship settings (3–6). In addition, patient-reported outcome studies suggest that many patients prefer approaches that avoid permanent visible tattoos, and some are willing to accept additional inconvenience or cost to do so (4).

To mitigate these concerns, tattoo-less or tattoo-sparing workflows have been increasingly investigated, including surface-guided radiotherapy (SGRT) based on optical surface imaging (7–10). A recent scoping review summarized multiple alternatives to dark ink tattoos, with SGRT being the most frequently evaluated approach among the included studies (7). Randomized crossover evidence in breast radiotherapy suggests that surface-guided positioning can reduce systematic and random setup errors compared with conventional tattoo- or laser-based workflows, while maintaining clinically feasible setup times (8). Other strategies—such as invisible ink tattoos, fluorescent tattoos, and temporary markings (e.g., henna or organic tattoo seals)—have also been proposed to reduce cosmetic permanence while preserving landmark utility (4, 12–14). Nevertheless, removal needs may persist for patients who already have permanent radiotherapy tattoos or other iatrogenic cutaneous markers. These conventional setup tattoos are typically small and monochromatic, and most commonly use black ink (4), and small controlled studies have compared removal approaches, such as Q-switched Nd:YAG laser vs. punch excision, for these tattoo sites (2). In routine clinical practice, ink selection may vary by institutional workflow and visibility considerations; while black (and sometimes dark blue) pigments are generally used, red pigments may also be employed occasionally to accommodate local simulation preferences, skin-tone contrast, or patient-specific considerations. Consequently, investigating removal modalities that can maintain consistent efficacy across a spectrum of pigment colors remains clinically relevant, even when the target marker is typically monochromatic.

Laser-based treatment remains a commonly used standard for tattoo removal, including Q-switched nanosecond systems and, more recently, picosecond devices (15–17). Because laser tattoo removal relies on wavelength-dependent optical absorption and subsequent pigment fragmentation, treatment response can vary substantially with pigment color and composition, deposition depth, and skin type (15, 16, 18–21). Multicolor tattoos often require wavelength-specific strategies and multiple sessions to achieve clinically meaningful clearance (15–17, 21). Adverse events such as blistering, hypo-/hyperpigmentation, textural change, scarring, allergic reactions, and paradoxical darkening have also been reported, depending on clinical context and pigment composition (16, 17, 22–24). Collectively, these limitations motivate continued interest in alternative modalities that are less constrained by pigment-specific optical properties and wavelength selectivity (15, 16).

High-intensity focused ultrasound (HIFU) is a non-optical energy modality that delivers acoustic energy to a defined focal region, and high-frequency implementations have been explored for dermal interventions (11, 25–28). In the context of tattoo treatment, 20-megahertz (MHz) high-frequency HIFU has been proposed as a non-optical approach because localized tissue effects are produced through acoustic energy deposition rather than selective optical absorption by the tattoo pigment (11, 25, 26). Accordingly, the treatment does not require selection of an optical wavelength matched to the absorption spectrum of each pigment. Preclinical studies in skin equivalents and animal models have reported that 20 MHz HIFU can generate small, localized thermal injury zones (TIZs) within superficial skin layers, with TIZ geometry and focal confinement influenced by transducer design and frequency (11, 26, 27). In minipigs, a 12-week local tolerance study of a 20 MHz HIFU system reported controllable, dose-dependent local responses with recovery over time under the evaluated conditions, supporting continued translational investigation (27). A human exploratory study further investigated 20 MHz HIFU for tattoo removal and reported clinical responses across different pigment colors, while emphasizing the need for continued mechanistic and safety characterization (25). Histological analyses of HIFU-induced TIZs have also been discussed in the broader therapeutic ultrasound literature, underscoring the value of tissue-level validation when interpreting proposed mechanisms (26, 27, 29).

For superficial dermal targets such as tattoo pigment, focal precision is a key consideration to maximize efficacy while minimizing collateral damage. Compared with lower-frequency ultrasound approaches designed for deeper tissue penetration, high-frequency (e.g., 20 MHz) transducers can be engineered for superficial targeting using a strong focusing geometry. For instance, a transducer with a low F-number (F# ≈ 0.75; a parameter indicating a wide convergence angle that allows the beam to focus sharply) is designed to concentrate acoustic energy into a highly confined target volume. Based on analytical modeling and fiber-optic hydrophone mapping in water, such devices have been reported to produce a tightly confined −6 dB focal zone (11, 25, 26). This zone defines the core acoustic hotspot where the effective pressure remains at or above 50% of its peak value, representing the primary region capable of inducing thermal coagulation. The physical dimensions of this hotspot are exceptionally small: the axial depth of focus (the vertical extent of the TIZ along the beam path) is on the order of ∼0.4–0.5 mm, and the lateral focal diameter (the horizontal width across the beam) is approximately ∼80 *μ*m. While these precise metrics are derived in water, actual focal dimensions *in vivo* may vary slightly depending on tissue attenuation (the natural absorption and scattering of sound by skin structures) and acoustic coupling conditions. Skin is a stratified tissue rather than a homogeneous acoustic medium. A prior study on 20–30 MHz measurements has shown that the epidermal and dermal compartments differ in attenuation and related propagation properties, with contributions from keratin- and collagen-rich structures (30). Another prior study on high-frequency skin sonography further indicates that superficial skin layers are acoustically distinct and interface-sensitive (31). Nevertheless, this degree of focal confinement theoretically enables the localization of thermal injury strictly within the dermis, sparing adjacent non-target tissues (11, 26, 27).

Despite prior work, there remains a notable gap in the literature regarding comprehensive, integrated validation of handheld high-frequency HIFU systems. Conventional tattoo removal literature has largely focused on Q-switched and picosecond laser platforms, for which treatment response depends on selecting an optical wavelength according to the absorption characteristics of the target pigment, followed by pigment fragmentation and downstream biological clearance (15, 16, 24). In contrast, 20 MHz HIFU is a non-optical energy-based approach that may induce localized dermal coagulative injury through acoustic energy deposition without requiring optical wavelength–pigment matching (25–27, 29). Because this pathway differs from conventional photomechanical tattoo fragmentation, histology-linked validation and cautious mechanistic interpretation are essential. Prior studies have typically evaluated *ex vivo* acoustic properties or isolated clinical outcomes, but they have rarely linked physical TIZ characterization to *in vivo* histopathological tracking of the proposed ablation-extrusion mechanism, alongside objective, multispectral quantification of efficacy across diverse tattoo colors (11, 25–27). Therefore, the present study evaluates a handheld 20 MHz HIFU system using a multi-model preclinical framework. Specifically, we (i) characterize TIZ formation in *ex vivo* tissue (11, 27, 32), (ii) examine histopathological features of multicolor tattoo removal in rats to assess whether tissue responses are consistent with a hypothesized ablation–extrusion working model (27, 29), and (iii) quantify tattoo clearance and skin recovery in minipigs using objective multispectral imaging (25, 27, 33–35). Ultimately, this stepwise approach aims to provide mechanistically informed, histology-linked evidence on the clinical feasibility of handheld high-frequency HIFU as a broad-spectrum tattoo removal modality.

## 2 Materials and methods

### 2.1 HIFU system and transducer specifications

High-intensity focused ultrasound (HIFU) exposures were delivered using a handheld, high-frequency dermatologic ultrasound system (PearlShot, ECO DM LAB Co., Ltd., Cheongju, Republic of Korea) (Figure 1). The device incorporates a strongly focused, single-element piezoelectric transducer with a nominal center frequency of 20 MHz. The console provides electronic control over the acoustic output, supporting a maximum rated power of 6 W and programmable exposure durations up to 1,000 ms. For this study, a standardized single-shot setting of 6 W for 1,000 ms (6 J per shot) was used. This parameter set was selected to promote consistent TIZ formation under the applied coupling conditions.

**Figure 1:**
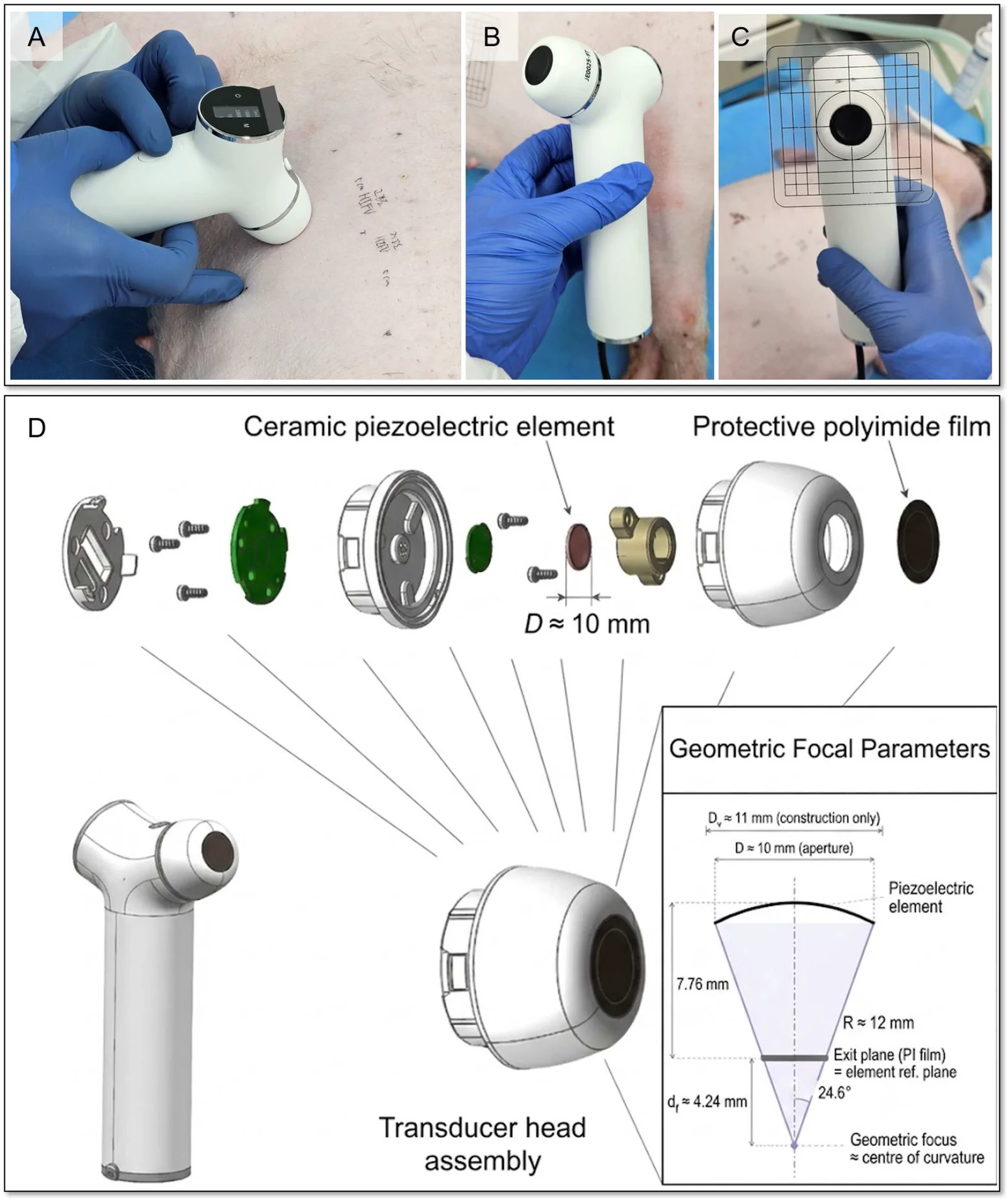
System configuration and provisional targeting methodology for the handheld high-intensity focused ultrasound (HIFU) device. **(A)** Representative macroscopic view of the handheld HIFU exposure on the skin surface. **(B)** Oblique view of the device handpiece. **(C)** View of the acoustic exit window fitted with the grid-marked provisional targeting film. **(D)** Exploded view of the handpiece head showing the transducer element and acoustic exit-window assembly, including the protective polyimide film. The inset in panel D illustrates the nominal geometric focal configuration, including the active aperture diameter (D≈10mm), virtual construction diameter $\left(D_{v}\approx 11\;\mathrm{mm}\right)$, radius of curvature (R≈12mm), PI-film exit-plane offset (7.76 mm), marginal-ray angle $\left(\approx 24.6^{\circ }\right)$, and nominal geometric focal distance from the exit plane $\left(d_{f}\approx 4.24\;\mathrm{mm}\right)$. The inset is not to scale and represents a geometry-based estimate.

Because the current handheld prototype (Figures 1A,B) did not include an integrated aiming camera or coaxial optical targeting guide to directly visualize the insonification spot on the skin surface, a provisional external reference was adopted to reduce gross placement variability. Specifically, the acoustic exit window of the device is inherently sealed with a protective polyimide (PI) film. To provide a spatial reference without obstructing or interfering with the acoustic beam path, a separate transparent film printed with a 5 mm × 5 mm grid pattern was utilized. This guiding film was designed with a central circular cutout corresponding to the acoustic aperture and was affixed to the exterior housing surrounding the exit window (Figure 1C). The surrounding grid served as a visual reference to align each shot with pre-marked skin coordinates. A coupling gel layer was applied between the device interface and the skin to ensure acoustic coupling and to minimize air gaps. While this structural approach was intended to improve the practical reproducibility of shot placement without compromising acoustic transmission, it may not guarantee exact microscopic coincidence between the external grid reference and the invisible acoustic focal axis under all coupling thicknesses, contact pressures, or surface curvatures. The device will be upgraded to improve targeting precision (e.g., by integrating an optical aiming module) and is under consideration for future studies.

To document the rationale for superficial targeting, focal placement was estimated using a two-step simplified ray model. First, a homogeneous-medium geometric construction was performed using the nominal transducer aperture and radius of curvature, with reference to previous analytical and experimental characterization of strongly focused 20 MHz HIFU transducers (11). The transducer head assembly (Figure 1D) was represented by an active aperture diameter of D≈10mm, a virtual diameter of $D_{v}\approx 11\;\mathrm{mm}$ used only for geometric construction, and a radius of curvature of R≈12mm. The corresponding marginal-ray half-angle relative to the central acoustic axis was approximately $24.6^{\circ }$. Because the tissue-facing PI-film exit plane was located approximately 7.76 mm from the transducer-element reference plane, the nominal geometric focal distance under the homogeneous-medium assumption was approximately 4.24 mm from the exit plane. Second, a refraction-aware planar multilayer estimate was performed to examine the potential influence of the intervening coupling and tissue interfaces. Ray angles were sequentially updated at each interface according to acoustic Snell's law, $\sin\theta_{i}/c_{i}=\sin\theta_{i+1}/c_{i+1}$, using nominal sound speeds and axial dimensions for the water path, PI film, coupling gel, epidermis, dermis, and subcutaneous fat, following established planar-layer ultrasound modeling (36). Under the nominal assumptions of a 0.5 mm coupling-gel layer, a 0.1 mm epidermal layer, and a 2.0 mm dermal layer, the rays were estimated to converge after propagating an additional 1.53 mm within the subcutaneous fat. This corresponded to an estimated convergence distance of approximately 4.13 mm from the tissue-facing PI-film exit plane and approximately 3.63 mm below the skin surface. These values represent geometry-based estimates under nominal planar-layer conditions and not direct measurements or full-wave simulations of the in-tissue acoustic pressure or intensity maximum.

### 2.2 *Ex vivo* tissue evaluation

To examine whether the handheld 20 MHz high-intensity focused ultrasound system could produce localized thermal effects at a reproducible depth, and to evaluate potential energy-dependent variations in TIZ dimensions, *ex vivo* experiments were performed using two distinct tissue-mimicking models. These included (i) a relatively homogeneous avian muscle model (chicken breast) to assess baseline acoustic energy deposition with minimal structural interference, and (ii) a full-thickness porcine skin tissue model (pork belly, comprising epidermal, dermal, and subcutaneous layers) to better approximate the acoustic attenuation, refraction, and structural complexity of human skin.

Fresh specimens were equilibrated at room temperature (25 ± 1 °C) and cut into blocks of approximately 50 × 50 × 10 mm^3^. Each block was positioned on a stable surface to minimize motion during exposure, and a coupling medium was applied to the interface to reduce air gaps. Ultrasound exposures were delivered using a fixed output setting of 6 W and a pulse duration of 1,000 ms per shot. To explore potential dose-response behaviors, the total delivered energy was incrementally varied from 9 to 60 J by adjusting the number of consecutive shots applied to the same target coordinates.

Following irradiation, the tissue blocks were vertically bisected along a plane intended to pass directly through the presumed focal axis. The exposed cross-sectional surfaces were inspected for macroscopic changes consistent with thermal injury, specifically localized whitening suggestive of coagulative necrosis. Images were captured using a digital microscope (Dino-Lite; AnMo Electronics Corp., New Taipei City, Taiwan) and analyzed via DinoCapture 2.0 software. For quantitative evaluation, the macroscopic TIZ dimensions were strictly defined relative to the acoustic beam trajectory: “depth” was defined as the axial extent of the TIZ parallel to the ultrasound beam path (representing vertical penetration), and “length” was defined as the lateral extent perpendicular to the beam path (representing horizontal spread across the focal plane). Measurements were obtained across replicate samples (*n* = 3) for each energy condition, with data summarized as mean ± standard deviation.

This *ex vivo* framework was intended to provide a controlled feasibility assessment of focal localization and energy-dependent TIZ formation under simplified conditions. Because *ex vivo* tissues inherently differ from living skin in terms of active perfusion, hydration levels, and dynamic mechanical properties, the observed TIZ boundaries and dimensions were interpreted as indicative of relative physical trends rather than as direct, absolute surrogates for *in vivo* tissue responses.

### 2.3 Animal models and preparation (rat and minipig)

All *in vivo* procedures were approved by the Institutional Animal Care and Use Committees (IACUC) of Yonsei University MIRAE Campus (Approval No. YWCI-202509-014-01) and Yonsei University Wonju College of Medicine (Approval No. YWC-250430-1). Experiments were conducted in accordance with applicable institutional and national guidelines for laboratory animal care and use. Two animal models were used to support complementary endpoints: eight-week-old female Sprague–Dawley rats (approximately 220 g) for histopathological assessment and 4–5 months old female Yucatan minipigs (approximately 15 kg) for macroscopic evaluation under skin conditions that more closely resemble human tissue.

Animals were anesthetized to minimize discomfort and reduce motion during high-intensity focused ultrasound procedures. Sprague–Dawley rats were anesthetized with 2% inhaled isoflurane. Hair over the dorsal treatment site was removed using an electric clipper, followed by a topical depilatory cream to reduce residual hair and facilitate acoustic coupling.

For Yucatan minipigs, anesthesia was induced by intramuscular administration of ketamine (50 mg/kg) and xylazine (4 mg/kg) and maintained with inhaled isoflurane (1–3 vol%). The designated skin regions were prepared by clipping only and gentle cleansing with sterile gauze. Chemical depilatory agents were not used in the minipig model to reduce the likelihood of superficial irritation or erythema that could interfere with subsequent macroscopic and histological assessments. Animals were monitored during anesthesia in accordance with local veterinary practice.

### 2.4 Tattooing procedure and HIFU treatment protocol

To support clinical relevance and procedural reproducibility of the iatrogenic dot-tattoo model, a radiation oncology specialist with experience in radiotherapy skin marking participated in the tattooing procedures. In Sprague–Dawley rats, an exploratory parameter-finding phase was performed on the lateral flank to refine a dot-matrix tattoo format that could approximate clinical point markers. Tattooing was performed using a commercially available DIGITAL-HAND-R permanent makeup machine (Bomtech Electronics Co., Ltd., Seoul, Republic of Korea) fitted with a sterile, single-use R-type needle cartridge. Needle diameter (0.18 vs. 0.40 mm), device speed (levels 1–3), and contact duration (1, 3, or 5 s per dot) were varied to identify a practical and reproducible setting. Based on this exploratory phase, a standardized dot-tattoo condition (0.40 mm needle, 3 s contact time, speed level 1) was selected for subsequent *in vivo* evaluation, and dot tattoos were placed on the rat dorsum (back). Commercial tattoo pigments (Beauty Equipment MANHATTAN) were used, including red, black, and blue pigments (Figure 2A).

**Figure 2:**
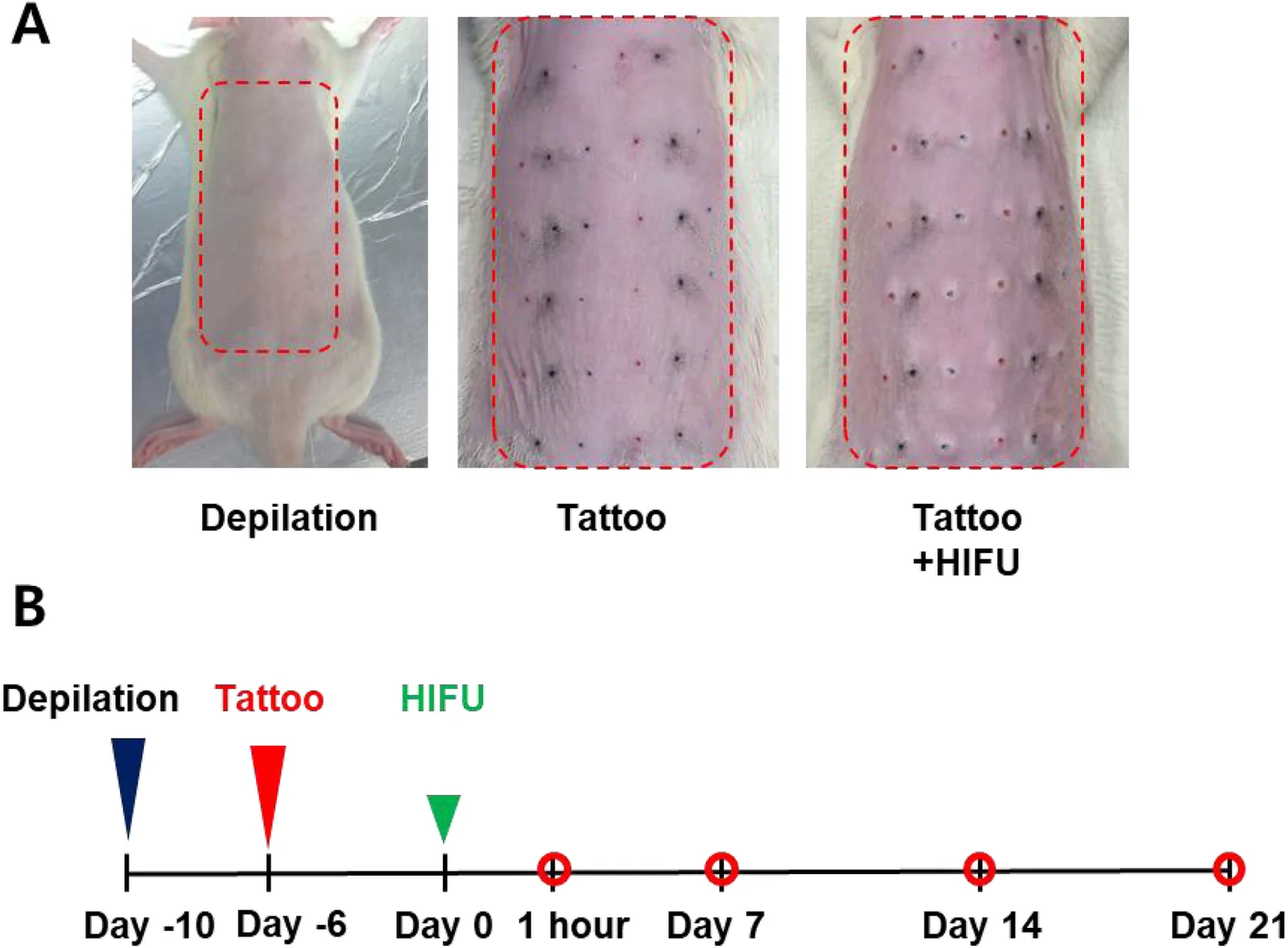
Experimental workflow and temporal assessment protocol for the *in vivo* rat tattoo model. **(A)** Representative macroscopic progression of the rat dorsal skin: post-depilation (left), following multicolor tattooing (middle), and immediately after 20 MHz HIFU irradiation (right). Red dashed boxes denote the targeted treatment regions. **(B)** Schematic of the sequential experimental timeline. The protocol outlines the intervals for skin stabilization post-depilation (4 days) and post-tattooing (6 days) prior to HIFU exposure, followed by the predefined temporal endpoints (1 h and Days 7, 14, and 21) for subsequent histopathological evaluation.

For the Yucatan minipig model, a feasibility-oriented longitudinal imaging design was adopted using a single female Yucatan minipig. This component was not intended to serve as a statistically powered efficacy experiment, but rather to evaluate whether serial multispectral three-dimensional (3D) imaging could capture acute and chronic surface-weighted responses after 20 MHz HIFU exposure under skin conditions that more closely approximate human cutaneous architecture than small-animal skin. Accordingly, no formal sample-size calculation was performed for the minipig arm. Three pigment colors (red, black, and blue) were symmetrically tattooed on both sides of the abdomen within an approximate 20 × 40 cm^2^ field per side (Figure 3). To reduce overlap of local inflammatory responses and facilitate longitudinal within-site assessment, tattoo dots were arranged with approximately 3 cm spacing. Because only one minipig was used, the resulting data were interpreted strictly as descriptive within-subject trajectories, and no inferential claims regarding treatment efficacy, reproducibility, or between-animal generalizability were made from this model.

**Figure 3:**
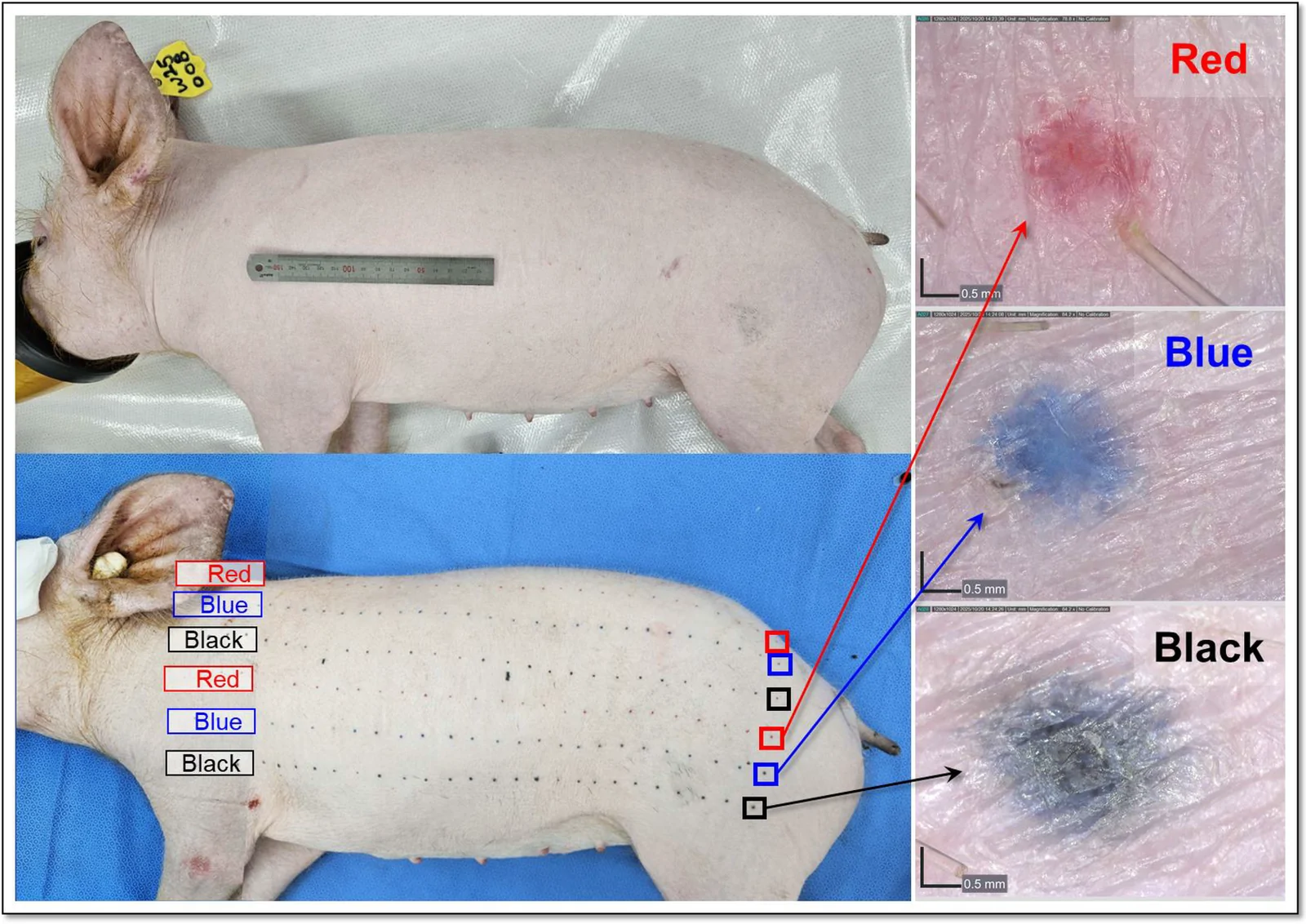
Macroscopic overview of the *in vivo* multicolor tattoo model in the Yucatan minipig.

Across all models, a 7-day stabilization period was strictly observed between the tattooing procedure and the subsequent HIFU exposure (Figure 2B). This interval was intended to ensure that acute mechanical irritation from the tattooing needles would not confound the post-treatment macroscopic assessments. Acoustic energy was delivered using a standardized 20 MHz HIFU exposure protocol. The device was operated at a manufacturer-reported acoustic output of 6 W for a 1,000 ms continuous-wave exposure. The 6 W value represented the time-averaged total acoustic output power measured in degassed water at 25 °C using a radiation force balance (UPM-DT-50SP, Ohmic Instruments), rather than the electrical input power or energy deposited at the focus. On this basis, each exposure corresponded to a nominal emitted acoustic energy of 6 J, and 10 repeated exposures corresponded to a cumulative nominal emitted acoustic energy of 60 J per treatment site. These values do not represent the energy absorbed or deposited within the tissue focal region. Each treatment site received 10 consecutive shots, resulting in a cumulative emitted acoustic energy of 60 J per treatment site. These values represent emitted acoustic energy characterized in water and not the energy absorbed or deposited within the tissue. A water-soluble ultrasonic gel was applied at the skin–device interface to reduce air gaps and maintain acoustic coupling. Spatial alignment for each treatment site was achieved using the provisional grid-based external reference affixed around the acoustic exit window. Across *in vivo* experiments, HIFU exposure was performed after a stabilization interval following tattooing to reduce confounding from acute needle-induced irritation.

### 2.5 Histopathological assessment in the rat model

To evaluate the microscopic tissue alterations and the temporal progression of wound healing, Sprague-Dawley rats were euthanized at the predefined histopathological endpoints (1 h and Days 7, 14, and 21 post-treatment) by gradual-fill carbon dioxide exposure at a displacement rate of 30% of the chamber volume per minute. Treated skin samples were subsequently harvested and fixed in 10% neutral buffered formalin, embedded in paraffin, and sectioned at a thickness of 4 *μ*m. Hematoxylin and Eosin (H&E) staining was performed to assess general morphological changes, inflammatory infiltration, and to delineate the boundaries of the TIZs. Additionally, Masson's Trichrome (MT) staining was utilized to evaluate dermal collagen remodeling and the spatial distribution of residual tattoo pigments. Stained histological slides were captured using a high-resolution optical microscope (DM2500; Leica, Wetzlar, Germany), and TIZ morphometric parameters were quantitatively measured on the histological sections using ImageJ software (version 1.53; NIH, Bethesda, MD, USA). Specifically, TIZ depth was defined as the maximum axial extent from the epidermal surface to the deepest histologically identifiable boundary of thermal injury, and TIZ length was defined as the maximum lateral extent of the histologically identifiable TIZ parallel to the skin surface.

### 2.6 Quantitative evaluation using multispectral imaging in the minipig model

In the Yucatan minipig model, tattoo-pigment–related surface changes and skin recovery were evaluated longitudinally using a multispectral 3D skin imaging system (Antera 3D, software version 3.1.16; Miravex Ltd., Dublin, Ireland). The system uses 24 light-emitting diodes and seven color channels spanning from red to blue across the visible spectrum. Multiple images acquired under directional illumination are processed using spatial and spectral reflectance analysis to generate CIELAB color and chromophore-related maps, while skin-surface topography is reconstructed using a proprietary shape-from-shading/photometric-stereo–based algorithm (33, 34). At each visit, the camera was placed in contact with the skin using a disposable cover, and images were acquired using consistent device settings and the manufacturer-recommended workflow. Because the spectral and 3D reconstruction algorithms are proprietary and visible-light based, the resulting measurements were interpreted as surface-weighted, algorithm-dependent descriptors rather than as direct depth-resolved measurements of tattoo pigment distribution or the acoustic focal zone. This distinction is important because visible-light penetration in skin is wavelength-dependent and largely confined to superficial tissue layers (37). Accordingly, the minipig imaging readouts were used as quantitative descriptors of surface recovery rather than as direct surrogates for the in-tissue acoustic focus. Although the Antera3D platform provides a broad range of device-generated outputs, the present study focused on a predefined subset of endpoints most directly related to tattoo clearance and skin recovery to preserve interpretability in this exploratory longitudinal dataset without over-complicating the model. To objectively monitor tissue responses, image analysis extracted specific variables across four parameters: color ($L^{*}$ average, $\Delta E_{ab}^{*}$), pigmentation (hyper-concentration), redness (maximum), and volume (volume in mm^3^ for acute analyses, mean height in mm for chronic analyses).

The *in vivo* imaging protocol was categorized into four analytical directions: acute response comparison, chronic response comparison, color-dependent chronic response, and dressing-dependent response. For the acute response comparison, a single HIFU-treated normal-skin site and two black tattoo sites were followed at pre-treatment (Pre-Tx), 15, 25, 35, 45, and 65–75 min. For the chronic response comparison, normal skin and a black tattoo site were followed longitudinally at Pre-Tx, Day 4, 25, 60, 73, and 98. For the color-dependent chronic response, red and black tattoos were recorded at Pre-Tx, an early post-treatment (Post-Tx) time point (∼37 min), and Day 101, whereas blue tattoos were recorded at Pre-Tx, Day 4, and Day 105. The slight difference in follow-up timing reflected a deliberate scheduling adjustment to avoid transient, rearing-related superficial abrasions that could confound interpretation of multispectral images. For the dressing-dependent response, a black tattoo site was divided into no-dressing and hydrocolloid-covered regions and followed at Pre-Tx, Post-Tx, Day 7, and Day 14. In the covered condition, a hydrocolloid dressing (DuoDERM ExtraThin; ConvaTec, Deeside, UK) was applied immediately after treatment.

To accommodate differences in the shape and extent of macroscopic tissue responses across experimental groups, regions of interest (ROIs) were tailored to the visible response boundary at each treated site and then kept constant across all longitudinal time points within that site. Because ROI size and shape varied among sites and analytical groups, Antera 3D-derived readouts were interpreted primarily as within-site temporal trajectories rather than as formal between-site comparisons.

In the acute analysis, colorimetric measurements were obtained using freehand polygonal ROIs (normal: 27.8 mm^2^; Case 1: 9.6 mm^2^; Case 2: 18.2 mm^2^), whereas redness and volumetric measurements were obtained using rectangular ROIs (normal: 10.0 mm × 8.3 mm; Case 1: 9.7 mm × 8.1 mm; Case 2: 8.5 mm × 7.5 mm). In the chronic comparison, all channels were analyzed using fixed polygonal ROIs (normal skin: 105.7 mm^2^; tattooed skin: 28.2 mm^2^). In the multicolor comparison, fixed rectangular ROIs were assigned to each tattoo color (red: 7.5 mm × 7.3 mm; black: 7.7 mm × 6.2 mm; blue: 6.4 mm × 7.1 mm). In the wound-care analysis, color metrics were extracted using polygonal ROIs (no dressing: 9.6 mm^2^; hydrocolloid: 18.2 mm^2^), whereas redness and topographic measurements were obtained using rectangular ROIs (no dressing: 9.7 mm × 8.1 mm; hydrocolloid: 8.5 mm × 7.5 mm).

Image analysis settings were held constant within each site across visits. Redness was quantified using hyper-concentration mode with a threshold of 15.37, pigmentation using hyper-concentration mode with a threshold of 23.91, and volume/topography using elevation mode with a 3 mm filter and a threshold of 0.05 mm; mean height was reported in millimeters.

Because the Antera 3D pigmentation and redness outputs are based on absorption-sensitive chromophore algorithms, these parameters were interpreted cautiously in tattooed sites containing exogenous inks. Likewise, topographic height trajectories were treated primarily as descriptive surface readouts rather than as definitive markers of re-crusting, because the observed sub-millimeter variations may be influenced by wound-surface condition, retained or detached eschar, imaging geometry, and contact conditions at the time of acquisition. For example, the Day 25 images in the chronic comparison were acquired under different wound-surface conditions: in the normal multi-spot region, eschars remained present at all four insonified zones, with only one site showing detachment at the time of imaging, whereas the tattooed skin was imaged after eschar detachment.

### 2.7 Blinding and bias control

To reduce assessment bias, investigators performing histological slide review, Antera 3D image processing, and quantitative extraction were blinded to treatment condition, pigment color, and group labels during endpoint evaluation whenever feasible. Measurements were performed independently by three blinded observers, and observer-level results were averaged to obtain final values for downstream summaries and, where applicable, statistical testing. In the minipig arm, formal between-animal randomization was not possible because only one animal was used. Therefore, bias control relied on a predefined within-subject layout, consistent imaging settings within each site across follow-up, avoidance of imaging time points affected by non-protocol skin injury, and blinded image processing.

In the minipig model, a within-subject design (multiple colors and/or dressing conditions within the same animal and anatomical region) was used to help control for inter-animal variability in baseline skin tone and tissue properties. Imaging time points affected by non-protocol skin injury were avoided as described above to reduce artifact-driven bias.

### 2.8 Statistical analysis

Quantitative data were analyzed in GraphPad Prism (version 9.3.1; GraphPad Software, San Diego, CA, USA) and are presented as mean ± standard deviation (SD) unless otherwise noted. Statistical testing was applied only to datasets with an experimental structure sufficient to support inferential analysis. *Ex vivo* TIZ measurements were analyzed across replicated energy conditions, and rat histopathology-derived morphometric measurements were analyzed across predefined post-treatment time points using site-level values averaged across blinded observers.

For the *ex vivo* morphometric evaluations, dimensional comparisons across different delivered energies and tissue substrates (avian vs. porcine models) were performed using a two-way analysis of variance (ANOVA) followed by Bonferroni's multiple comparisons test.

For the rat histopathology-derived morphometric analysis, site-level values averaged across three blinded observers were used for statistical testing. Temporal changes in pooled TIZ length and depth across the four observation time points were evaluated using the Kruskal–Wallis test, followed by Dunn's multiple comparisons test for *post hoc* pairwise comparisons.

For the *in vivo* minipig model, Antera 3D-derived outcomes were analyzed exclusively as descriptive longitudinal trajectories. Because the minipig component used a single animal and was not designed for between-animal replication, randomized group comparison, or formal hypothesis testing, no inferential *P*-values were calculated or interpreted for minipig imaging endpoints. These outcomes were therefore used only to describe within-site temporal patterns and concordance across surface-weighted imaging metrics.

## 3 Results

### 3.1 *Ex vivo* validation of focal precision and energy-dependent TIZ formation

Following HIFU irradiation, localized macroscopic whitening, consistent with thermal coagulative necrosis, was observed along the presumed acoustic focal axis in both the relatively homogeneous avian muscle and full-thickness porcine tissue models (Figures 4A,B). Quantitative morphometric analysis (Figure 4C) demonstrated an energy-dependent expansion of TIZ dimensions across the 9–60 J range. Two-way ANOVA confirmed a significant main effect of delivered energy on both TIZ length and depth (both *p* < 0.001). For lateral expansion (length), both models showed comparable overall trends, with no significant main effect of tissue type (*p* = 0.76) and no significant interaction between energy and tissue type (*p* = 0.19). This indicates that both models exhibited comparable lateral expansion patterns governed primarily by the delivered energy dose.

**Figure 4:**
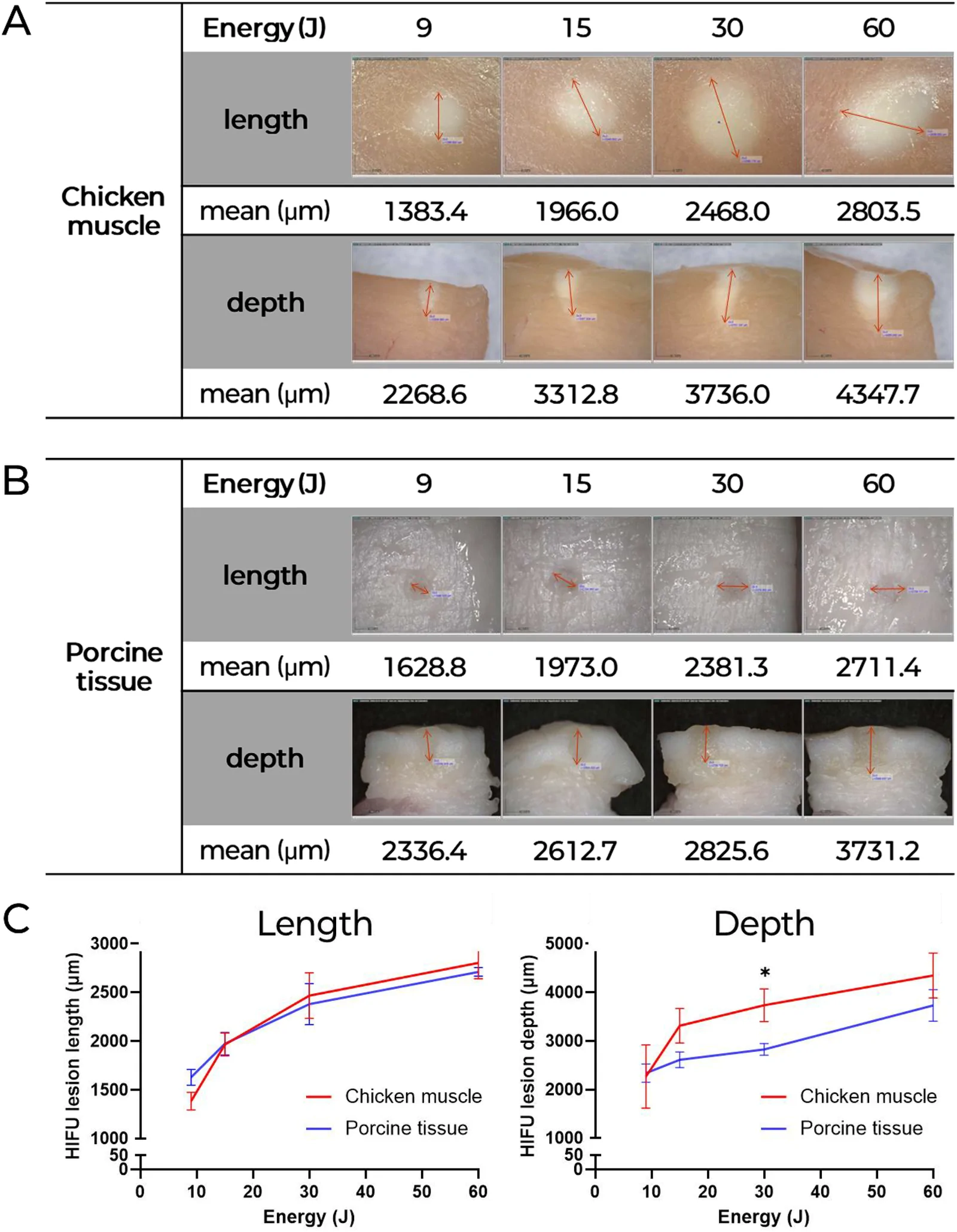
*Ex vivo* energy-dependent changes in thermal injury zone (TIZ) geometry in avian muscle and full-thickness porcine skin tissue models. **(A)** Representative macroscopic surface views (upper row) and corresponding cross-sectional views (lower row) of localized TIZs in the avian muscle (chicken breast) model at delivered energies of 9, 15, 30, and 60 J. **(B)** Representative macroscopic surface views (upper row) and corresponding cross-sectional views (lower row) of localized TIZs in the porcine tissue model at the same energy levels. **(C)** Quantitative comparison of TIZ length and depth as a function of total delivered energy in both *ex vivo* models, shown as mean ± SD (*n* = 3 per condition). The asterisk denotes the significant between-tissue comparison identified by Bonferroni's multiple comparisons test following two-way ANOVA (* adjusted *p* < 0.05).

In contrast, axial expansion (depth) was influenced by tissue type in addition to delivered energy. Two-way ANOVA revealed that TIZ depth was significantly affected by both delivered energy (*p* < 0.001) and tissue type (*p* = 0.002), whereas the interaction between energy and tissue type was not significant (*p* = 0.15). Although baseline depth at 9 J was similar between the two models, the porcine tissue model showed overall shallower axial extension than the avian muscle model at higher energy levels. *Post hoc* multiple comparisons further identified a significant between-tissue difference at 30 J (adjusted *p* < 0.05). Collectively, these *ex vivo* morphometric findings suggest that while lateral focal confinement remained comparable across tissue types, axial TIZ depth was additionally influenced by tissue structure, with the full-thickness porcine model showing relatively constrained downward extension.

### 3.2 *In vivo* macroscopic and histopathological findings consistent with transepidermal clearance of multicolor pigment in the rat model

To characterize the gross course of healing after HIFU treatment, multicolor dot tattoos on the rat's dorsal skin were followed macroscopically over time (Figure 5). At baseline (one week after tattooing), all tattoo dots were well demarcated, without visible surrounding erythema or spontaneous fading. Approximately 1 h after treatment, each site showed localized surface whitening confined to the targeted area, accompanied by a transient perifocal elevation. During the subacute phase (Days 7–14), the initial whitening resolved and discrete crusts formed over the treated sites, with a grossly similar appearance across pigment colors. By Day 21, most crusts had spontaneously detached, and the treated sites showed a notable visible reduction in pigment intensity relative to the baseline. Mild residual ring-like perifocal changes remained visible at some sites, indicating incomplete gross normalization within the observation window.

**Figure 5:**
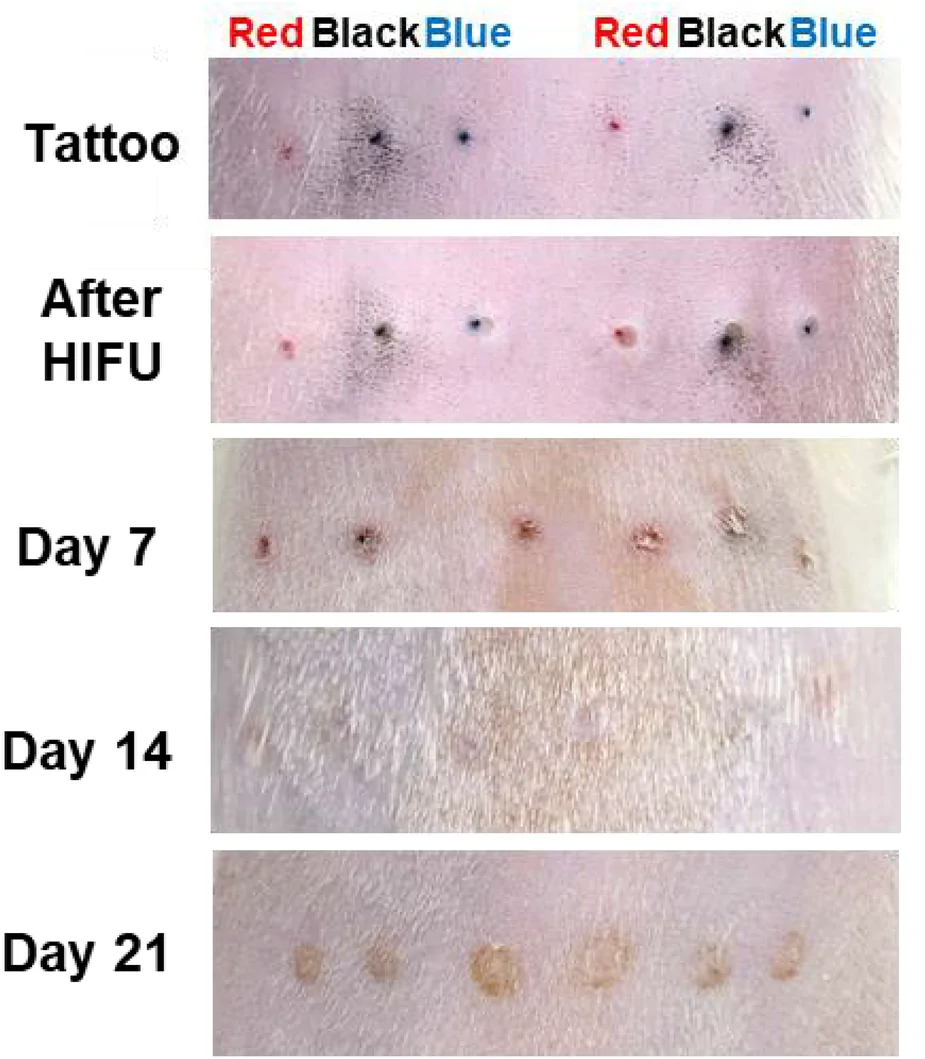
Longitudinal macroscopic healing trajectory of multi-color tattoos following a single-session 20 MHz HIFU exposure in the *in vivo* rat model.

Longitudinal histopathological evaluation demonstrated tissue changes consistent with progressive transepidermal pigment clearance (Figures 6A,B). In untreated control skin, tattoo pigments were densely aggregated within the upper-to-mid dermis without evident pre-existing inflammation. At 1 h after HIFU exposure, distinct TIZs were identified, showing localized coagulative change, interstitial edema, and early disruption of dense pigment clusters in the targeted dermal layer. During Days 7–14, the treated sites exhibited reactive tissue changes and an overlying epidermal crust. Within this interval, pigment-containing debris was frequently observed redistributed toward the dermo-epidermal junction and incorporated into the crust. By Day 21, following crust detachment, re-epithelialization and dermal collagen remodeling were evident, accompanied by a visible reduction in residual dermal pigment density.

**Figure 6:**
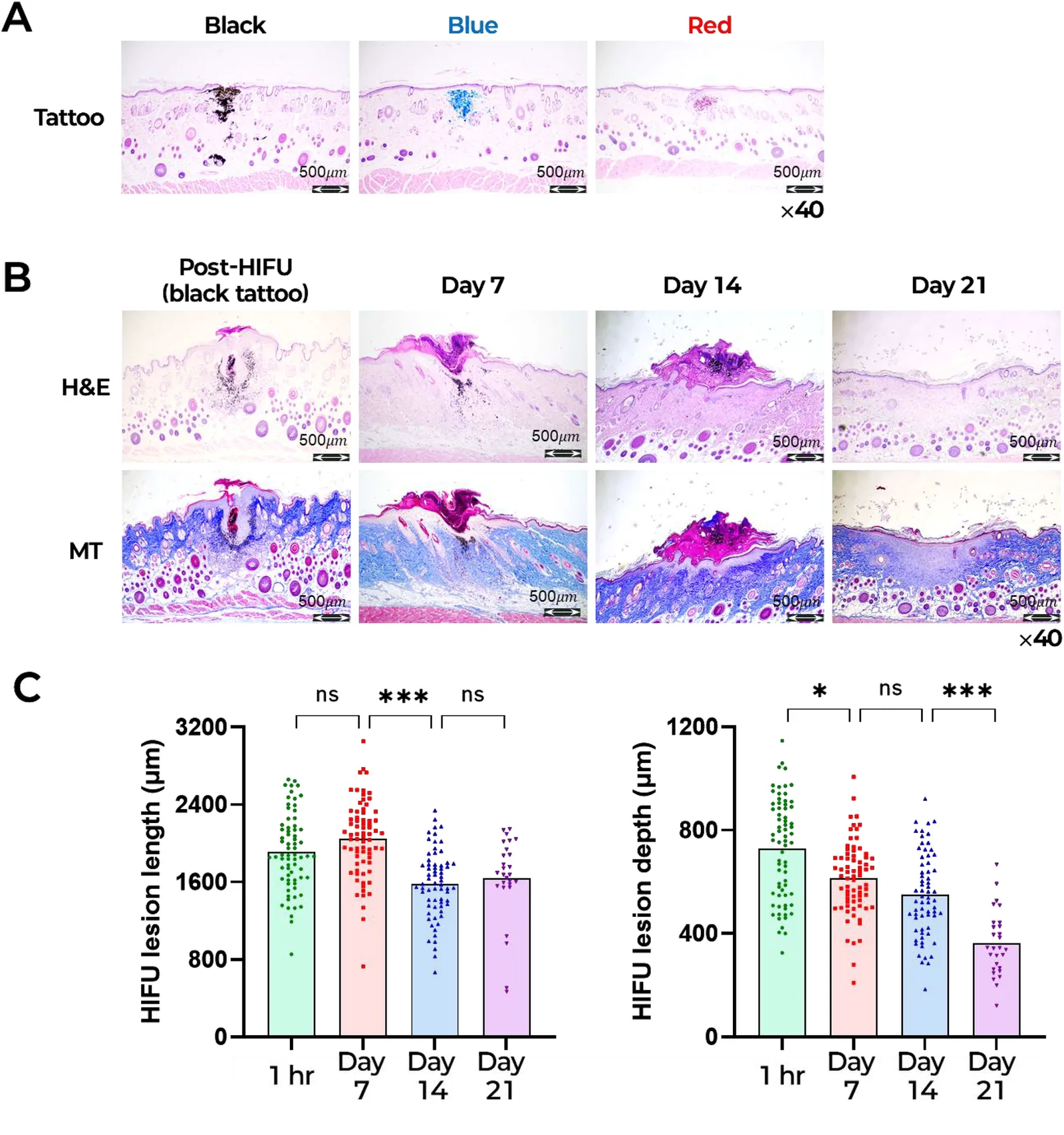
Longitudinal histopathological and morphometric evaluation after 20 MHz HIFU exposure to the tattooed sites on rat dorsal skin. **(A)** Tattoo-only controls (black, blue, and red), showing dermal pigment localization on hematoxylin and eosin (H & E) staining. **(B)** Representative post-treatment histology from black-tattoo sites, showing focal thermal injury, crust formation (Days 7–14), and re-epithelialization with dermal remodeling (Day 21) on H & E and Masson's trichrome (MT) staining (×40). **(C)** Temporal trajectories of TIZ length and depth, shown as mean ± SD from site-level values averaged across three blinded observers. Only the indicated pairwise comparisons are annotated: *Adjusted *p* < 0.05 and ***Adjusted *p* < 0.001, based on Dunn's multiple comparisons test following a Kruskal–Wallis test.

Because the gross and early microscopic healing responses were qualitatively similar across pigment colors, morphometric data were pooled to assess the overall temporal dynamics of the TIZs (Figure 6C). The number of analyzed sites at each time point was *n* = 70 (1 h), *n* = 73 (Day 7), *n* = 66 (Day 14), and *n* = 28 (Day 21). Kruskal–Wallis testing showed a significant overall effect of time on both TIZ length and depth (both *p* < 0.001). The mean TIZ length increased from 1,912 ± 398 *μ*m at 1 h to 2,047 ± 392.7 *μ*m at Day 7, and then decreased to 1,578 ± 353.7 *μ*m at Day 14 and 1,635 ± 435.2 *μ*m at Day 21. Dunn's multiple comparisons test showed that TIZ length was significantly smaller at Day 14 than at both 1 h and Day 7 (both adjusted *p* < 0.001), and was also significantly smaller at Day 21 than at Day 7 (adjusted *p* < 0.001), whereas the other pairwise comparisons were not significant.

In contrast, mean TIZ depth decreased progressively from 730.0 ± 195.5 *μ*m at 1 h to 615.1 ± 141.8 *μ*m at Day 7, 551.1 ± 163.2 *μ*m at Day 14, and 361.8 ± 124.9 *μ*m at Day 21. *Post hoc* testing confirmed that TIZ depth was significantly reduced at Day 7 (adjusted *p* = 0.02), Day 14 (adjusted *p* < 0.001), and Day 21 (adjusted *p* < 0.001) relative to 1 h. In addition, depth at Day 21 was significantly shallower than at both Day 7 and Day 14 (both adjusted *p* < 0.001), whereas the difference between Day 7 and Day 14 was not significant (adjusted *p* = 0.18). Overall, these longitudinal histopathological observations were consistent with a healing sequence characterized by early focal thermal injury, transient lateral expansion, progressive axial contraction, and crust-associated upward redistribution of pigment during tissue repair. The localization of pigment debris near the dermo-epidermal junction and within crusts was compatible with a proposed transepidermal extrusion pathway; however, the cellular basis of this process was not directly assessed in the present study.

### 3.3 *In vivo* multispectral quantification of cutaneous responses and healing trajectories in the minipig model

Acute cutaneous responses after a single 20 MHz HIFU exposure were assessed in one normal-skin site and two black tattoo sites (Figure 7). At baseline, tattooed sites exhibited lower lightness ($L^{*}$ average, 66.69–69.09) and higher total color difference ($\Delta E_{ab}^{*}$, 11.59–15.59) compared with normal skin ($L^{*}$ 78.42, $\Delta E_{ab}^{*}$ 1.76), consistent with the optical influence of dense black pigment. Following exposure, the two site types diverged in their early color trajectories. In normal skin, lightness decreased at 15 min [78.42 → 75.42 (−3.8%)], while $\Delta E_{ab}^{*}$ increased [1.76 → 3.28 (+86.5%)], indicating an acute deviation from baseline skin tone during the early post-treatment interval. In tattooed sites, lightness increased modestly by 15 min [Case 1: 69.09 → 69.63 (+0.8%); Case 2: 66.69 → 67.59 (+1.4%)], accompanied by a marked reduction in $\Delta E_{ab}^{*}$ [Case 1: 11.59 → 6.52 (−43.7%); Case 2: 15.59 → 9.72 (−37.7%)], indicating a rapid decrease in visible color contrast relative to baseline within the tattoo region. Thereafter (25–65 min), normal skin lightness gradually returned toward baseline (−1.5% at 65 min), whereas tattooed sites remained slightly elevated compared with baseline (+3.7% in Case 1 and +3.5% in Case 2 at 65 min). $\Delta E_{ab}^{*}$ remained around ∼3.0 in normal skin (2.99–3.10) and stabilized at lower-than-baseline levels in tattooed sites after the initial drop (Case 1: ∼6.48–6.68; Case 2: ∼10.57–11.76).

**Figure 7:**
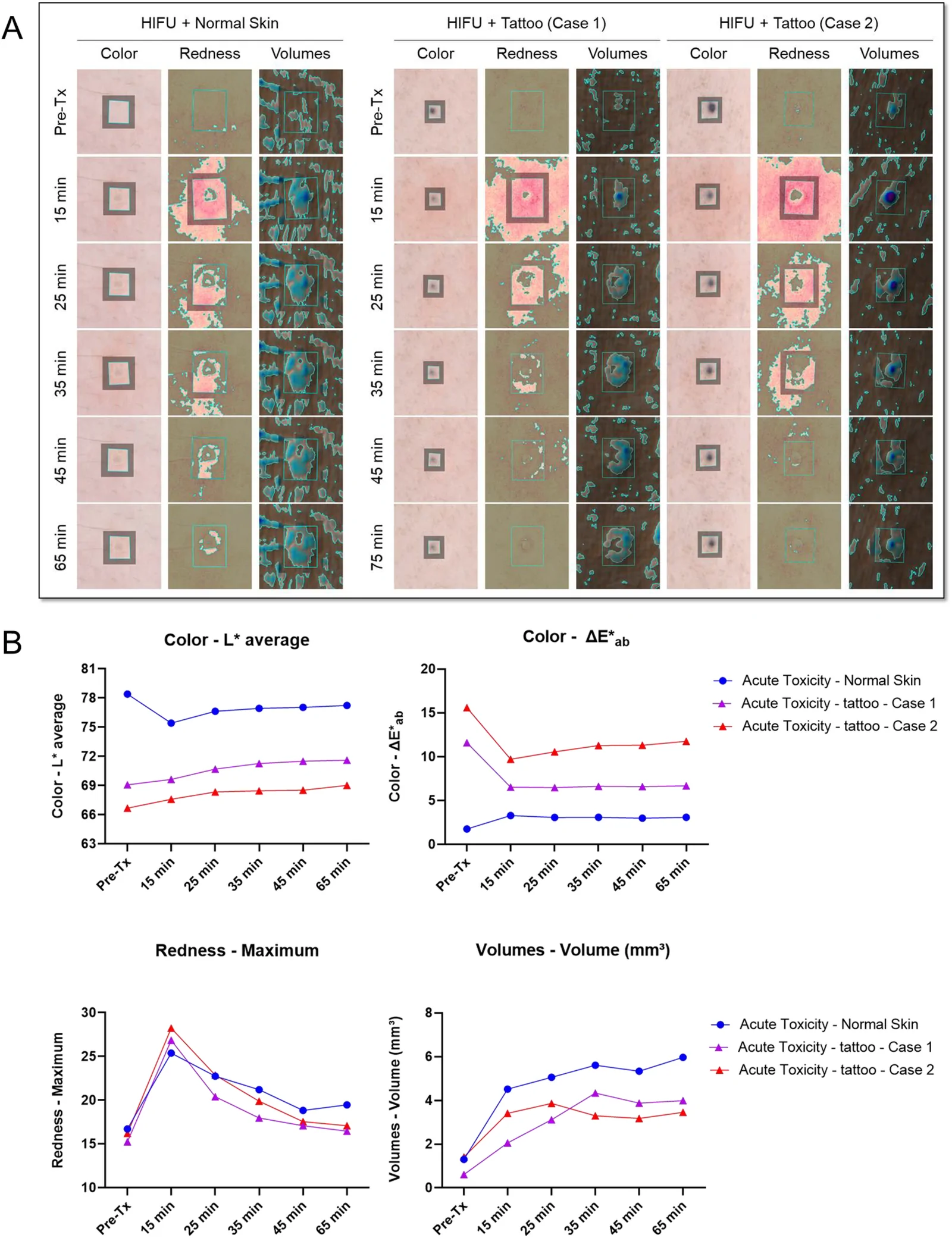
Comparative time-course analysis of acute cutaneous responses to 20 MHz HIFU irradiation in normal and tattooed minipig skin. **(A)** Representative multi-spectral three-dimensional (3D) surface images acquired by the optical imaging system (Antera3D) at baseline and subsequent follow-up time points in normal (single case) and tattooed (two cases) skin. **(B)** Quantitative temporal trajectories of colorimetric and topographical parameters: lightness ($L^{*}$ average), total color difference $\left(\Delta E_{ab}^{*}\right)$, maximum redness, and surface volume $\left(mm^{3}\right)$.

For vascular responses, maximum redness peaked at 15 min at all sites [normal: 16.72 → 25.40 (+51.9%); Case 1: 15.26 → 26.85 (+75.9%); Case 2: 16.23 → 28.24 (+74.0%)], then declined. Notably, normal skin remained elevated above baseline (+16.5%) at 65 min, whereas tattooed sites approached baseline (Case 1: +8.0%; Case 2: +5.3%) by 65 min.

Surface volume increased over the observation window in all sites, but the temporal profiles differed: normal skin showed a progressive increase up to 65 min [1.30 → 5.97 mm^3^ (+359.2%)], while tattooed sites showed different volumetric temporal profiles, with earlier peaks or plateaus [Case 1 peaked at 35 min: 0.61 → 4.33 mm^3^ (+610.9%); Case 2 peaked at 25 min: 1.41 → 3.86 mm^3^ (+173.8%)], with values remaining within ∼3.18–3.99 mm^3^ thereafter.

Chronic cutaneous recovery was tracked longitudinally in normal skin and black-tattooed skin over 98 days (Figure 8). Colorimetric trajectories showed an early deviation at Day 4 in both sites, exhibiting a marked decrease in lightness [$L^{*}$ average: normal 79.79 → 71.16 (−10.8%), tattoo 76.90 → 62.91 (−18.2%)] and an increase in total color difference [$\Delta E_{ab}^{*}$: normal 2.60 → 9.14 (+252.1%), tattoo 9.87 → 15.34 (+55.4%)] from pre-treatment (Pre-Tx). By Day 25, $L^{*}$ remained below baseline in both sites (normal 73.62, tattoo 74.43), while $\Delta E_{ab}^{*}$ stayed elevated (normal 8.88, tattoo 9.23). Thereafter, $L^{*}$ gradually returned toward baseline by Day 60 in both sites. At the final visit (Day 98), normal skin returned to near-baseline color levels ($L^{*}$ +0.5%, $\Delta E_{ab}^{*}$ −4.1%), whereas the tattooed skin showed a substantial reduction in $\Delta E_{ab}^{*}$ (−66.6%) and increase in $L^{*}$ (+1.8%). By the final visit, the tattooed site showed marked reduction in total color difference and recovery of lightness, approaching the threshold of visually perceptible contrast $\left(\Delta E_{ab}^{*}∼3\right)$.

**Figure 8:**
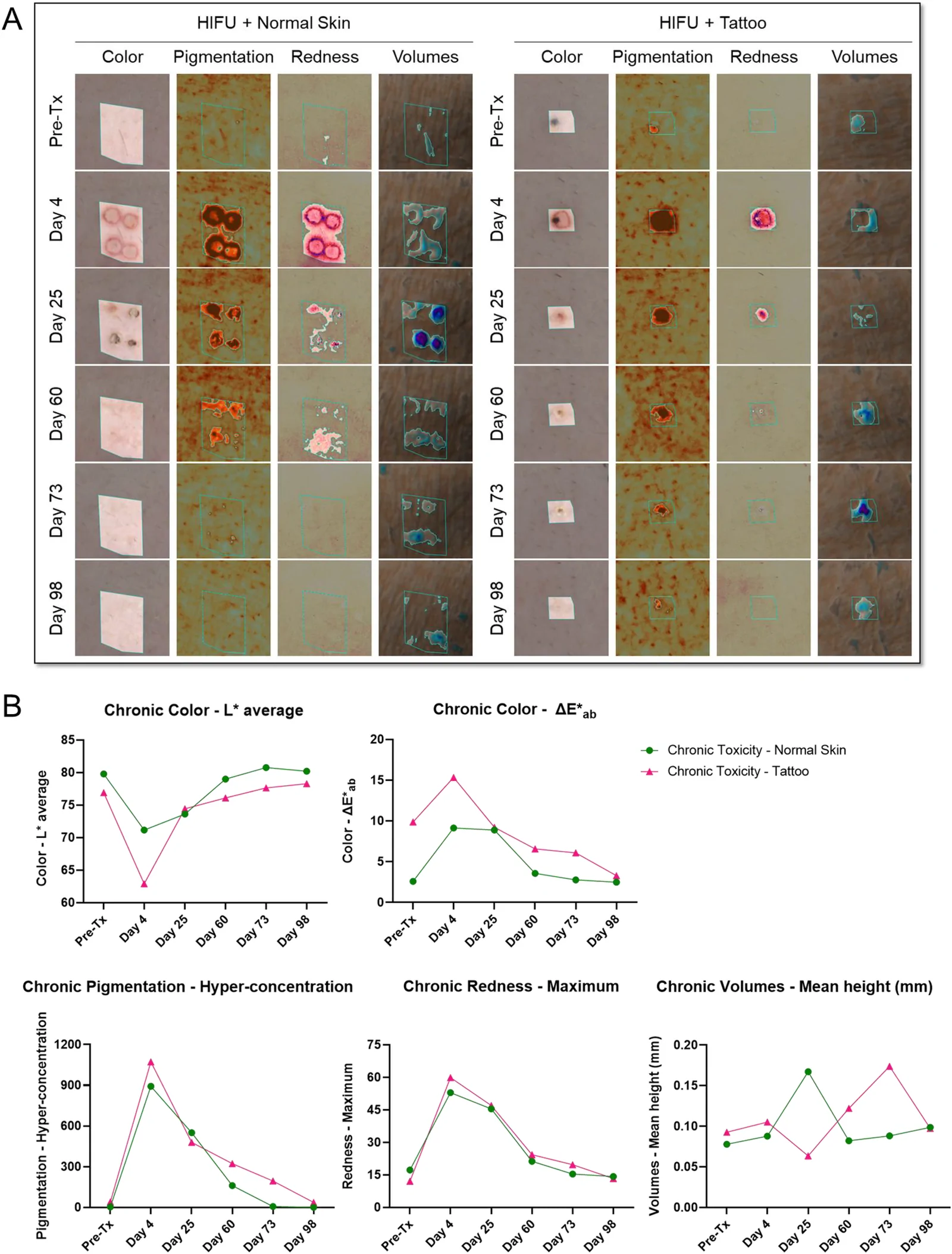
Comparative time-course analysis of chronic cutaneous responses and recovery after 20 MHz HIFU irradiation in normal and tattooed minipig skin. **(A)** Representative multispectral 3D surface images acquired by Antera3D at baseline and subsequent long-term follow-up (Day 4–Day 98) time points in normal (multi-spot) and tattooed (single-spot) skin. **(B)** Quantitative temporal trajectories of colorimetric and topographical parameters: lightness ($L^{*}$ average), total color difference $\left(\Delta E_{ab}^{*}\right)$, hyper-concentrated pigmentation, maximum redness, and mean surface height (mm).

Pigmentation and maximum redness peaked at Day 4 in both normal (multi-spot) and tattooed (single-spot) skin, then declined. Pigmentation rose from 3.25 to 892.73 in normal skin and from 40.87 to 1,072.61 in tattooed skin at Day 4, then decreased to near-baseline levels (0.12 and 36.74, respectively) by Day 98. Similarly, maximum redness increased from 17.26 to 52.96 (normal) and from 12.12 to 59.91 (tattoo) at Day 4, approaching baseline levels (14.30 and 13.32, respectively) by Day 98. Between Day 25 and Day 73, the tattooed site maintained higher localized pigmentation values than the normal site (normal 550.82 → 6.27, tattoo 479.57 → 194.11), aligning morphologically with the persistent central brownish dot-like residual feature observed within the tattoo ROI. A persistent, focal, brownish residual feature remained appreciable at the tattooed site but not in normal skin. Overall, both pigmentation and redness metrics in both groups recovered toward their respective baselines by Day 98.

Topographical recovery (mean surface height) remained within a sub-millimeter range across all visits. In normal skin, mean height increased from 0.078 mm (Pre-Tx) to 0.088 mm (Day 4) and peaked at 0.167 mm (Day 25), reflecting the physical presence of retained eschars, before decreasing to 0.099 mm by Day 98. In tattooed skin, where the primary eschar had already shed prior to the Day 25 measurement, mean height temporarily decreased from 0.105 mm (Day 4) to 0.064 mm (Day 25). In tattooed skin, mean height temporarily decreased at Day 25 and then showed re-elevation at Days 60–73 before returning near baseline by Day 98.

To assess whether the long-term response after 20 MHz HIFU was broadly consistent across different tattoo pigments, multispectral 3D imaging (Figure 9) was applied to red, black, and blue tattoo sites.

**Figure 9:**
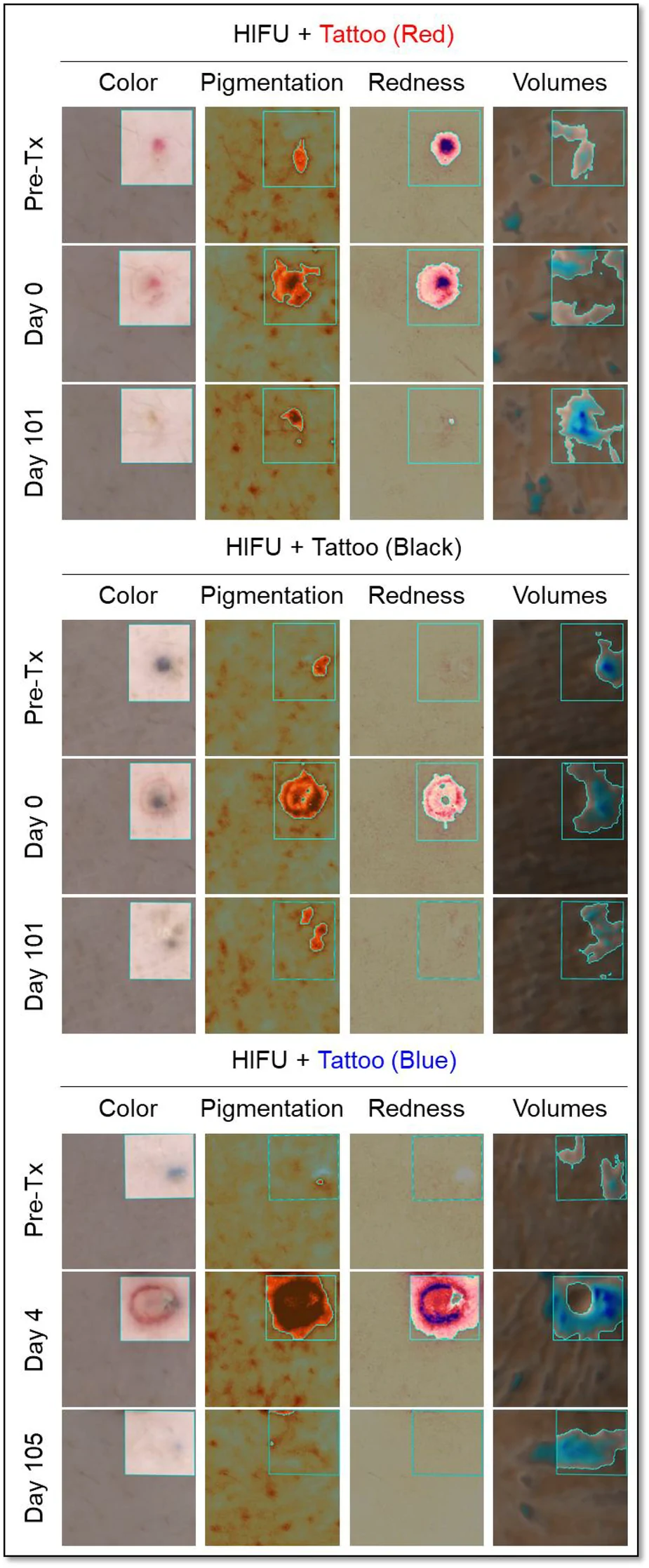
Representative long-term (>100-day) multispectral 3D surface images acquired by Antera3D, showing multicolor tattoo clearance and skin recovery following 20 MHz HIFU in minipigs.

Across all three colors, the color-channel metrics showed a convergent long-term trend toward reduced tattoo contrast and recovery of lightness. Specifically, $\Delta E_{ab}^{*}$ decreased at the final time point compared with baseline [red: 6.51 → 3.45 (−47.0%), black: 8.36 → 5.00 (−40.2%), blue: 5.69 → 3.46 (−39.2%)], while $L^{*}$ returned to near-baseline levels by Day 101–105 [red: 76.75 → 78.13 (+1.8%), black: 78.18 → 77.64 (−0.7%), blue: 77.84 → 78.47 (+0.8%)]. In the blue tattoo, the Day 4 state showed a pronounced transient change [$L^{*}$: 77.84 → 65.56 (−15.8%), $\Delta E_{ab}^{*}$: 5.69 → 12.35 (+116.8%)], which was comparable in magnitude to the Day 4 pattern observed in the chronic comparison (Figure 8), and then recovered to the baseline level by Day 105.

Pigmentation and redness exhibited large early elevations followed by gradual attenuation. At the final follow-up (Day 101 for red/black and Day 105 for blue), pigmentation hyper-concentration was higher than baseline across all colors [red: 35.89 → 88.04 (+145.3%), black: 55.56 → 67.76 (+22.0%), blue: 8.48 → 25.32 (+198.5%)]. By Day 101–105, redness trajectories for the black and blue tattoos approached low, near-baseline levels [black: 15.04 → 14.50 (−3.6%); blue: 13.98 → 14.62 (+4.6%)]. Conversely, the red tattoo exhibited a markedly large baseline redness value (82.97), which subsequently decreased to 17.92 (−78.4%) by Day 101. This exaggerated baseline reading may reflect spectral overlap between the red ink and the hemoglobin-sensitive redness algorithm.

The influence of early wound care was assessed by comparing no-dressing and hydrocolloid-covered tattoo sites over 14 days (Figure 10). Immediately after treatment (∼32 min), both conditions showed a modest increase in $L^{*}$ average [no dressing: 69.09 → 69.63 (+0.8%), hydrocolloid: 66.69 → 67.59 (+1.4%)] with a concurrent reduction in $\Delta E_{ab}^{*}$ [no dressing: 11.59 → 6.52 (−43.7%), hydrocolloid: 15.59 → 9.72 (−37.7%)]. By Day 7, both conditions showed lower $L^{*}$ values [no dressing: 69.09 → 59.70 (−13.6%), hydrocolloid: 66.69 → 56.94 (−14.6%)] and higher $\Delta E_{ab}^{*}$ [no dressing: 11.59 → 13.66 (+17.9%), hydrocolloid: 15.59 → 16.19 (+3.9%), respectively], consistent with an eschar-dominant visual phase in the serial images.

**Figure 10:**
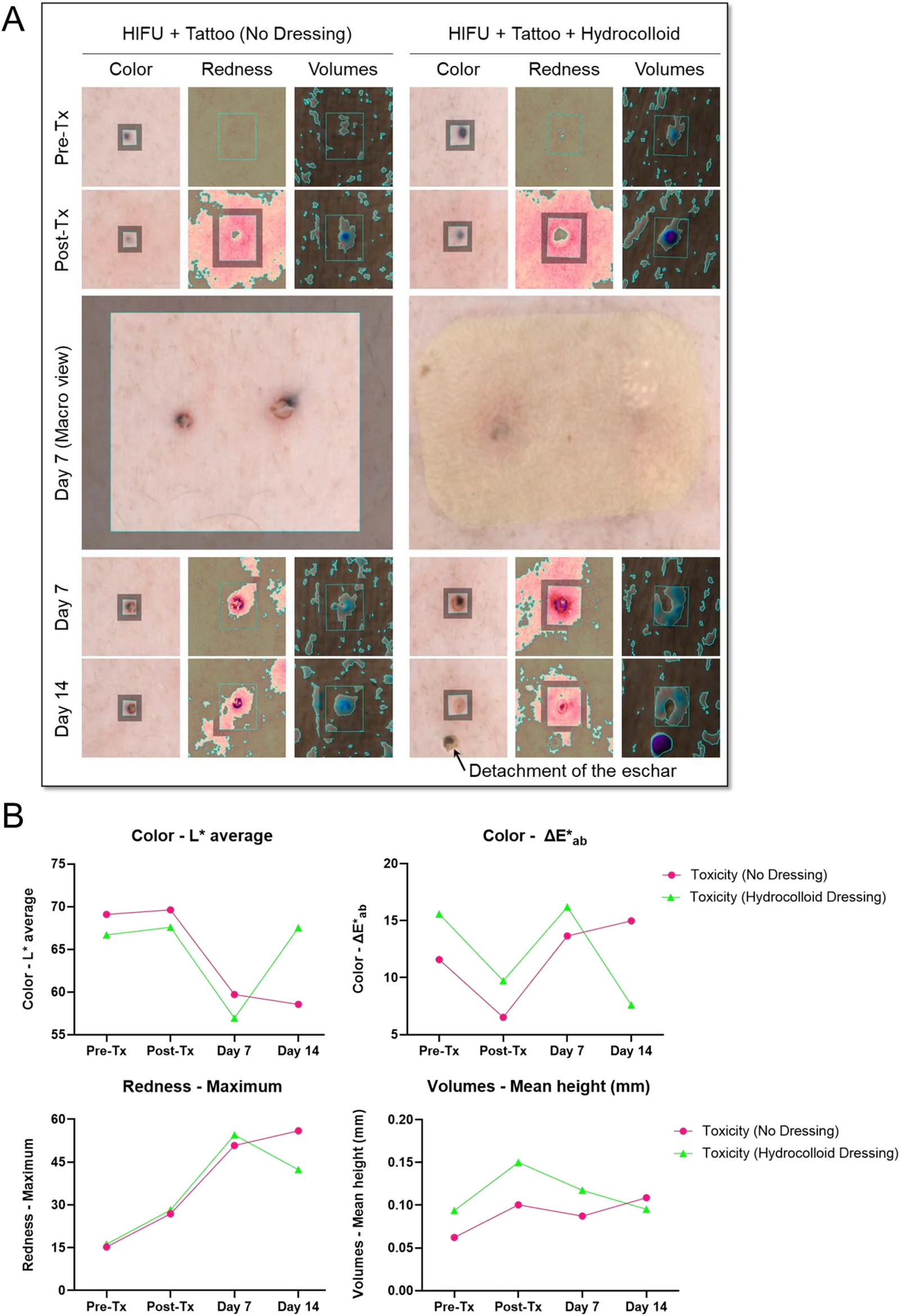
Influence of hydrocolloid dressing on early healing and eschar dynamics after 20 MHz HIFU irradiation in tattooed minipig skin. **(A)** Representative multispectral 3D surface maps acquired by Antera3D over a 14-day follow-up and a macroscopic photograph (showing the presence of the hydrocolloid patch at Day 7) in the no-dressing and hydrocolloid-dressing conditions. **(B)** Quantitative temporal trajectories of colorimetric and topographical parameters: lightness ($L^{*}$ average), total color difference $\left(\Delta E_{ab}^{*}\right)$, maximum redness, and mean surface height (mm).

At Day 14, trajectories diverged. In the hydrocolloid condition, $L^{*}$ returned close to baseline [66.69 → 67.53 (+1.3%)] with a lower $\Delta E_{ab}^{*}$ [15.59 → 7.59 (−51.3%)], coinciding with visual evidence of eschar detachment. In contrast, the no-dressing condition remained darker [$L^{*}$ 69.09 → 58.56 (−15.2%)] with a higher $\Delta E_{ab}^{*}$ [11.59 → 14.99 (+29.4%)], indicating delayed normalization within the observation window.

Vascular and topographical readouts were also directionally different by Day 14: maximum redness continued to rise in the no-dressing condition (Day 7 → Day 14: 50.82 → 55.98), whereas it decreased in the hydrocolloid condition (54.52 → 42.31). Mean surface height remained elevated without dressing (Pre-Tx → Day 14: 0.062 → 0.109 mm), while it returned near baseline with hydrocolloid coverage (0.094 → 0.095 mm).

## 4 Discussion

The *ex vivo* morphometric comparisons demonstrate that 20 MHz acoustic energy generates superficially confined TIZs. Although the geometric ray-tracing model estimated convergence at approximately 4.13 mm below the PI film surface under nominal assumptions, the visible TIZ depth at 9 J was shallower in both tissue models. This discrepancy likely reflects a combination of high-frequency attenuation, threshold-dependent visualization of coagulative injury, and potential underestimation introduced by sectioning geometry. As delivered energy increased, axial TIZ expansion became more pronounced, but this progression remained more limited in the layered porcine tissue model than in the homogeneous avian muscle model, suggesting that tissue architecture can constrain deeper thermal spread. Overall, these findings support the feasibility of generating localized superficial TIZs and highlight that the layered skin structure may provide a practical buffer against excessive deep propagation.

Building on the *ex vivo* feasibility findings, the rat model provides histological evidence that a single 60 J exposure can generate a superficially confined dermal injury that evolves through a wound-healing–linked clearance process. The sub-millimeter axial extent of the TIZ observed *in vivo* is directionally consistent with the expectation that perfusion-mediated heat removal and the complex, multi-layered architecture of living skin may further restrict thermal spread compared with non-perfused *ex vivo* substrates. It has been reported that the complex composition of the stratum corneum and epidermis introduces multiple tissue interfaces, which could increase acoustic attenuation, scattering, and refraction (30, 31). Furthermore, in a living biological system, acute physiological cellular responses—such as transient edema and subsequent alterations in tissue hydration and collagen density—might dynamically alter local acoustic properties over time (38, 39). The sharply demarcated coagulative morphology observed in the present study was compatible with a predominantly thermal tissue response. However, HIFU bioeffects are not necessarily exclusively thermal. Radiation-force–related stress, acoustic streaming, and cavitation-related bubble activity may contribute to localized mechanical effects, modify heat deposition, or influence lesion morphology (29, 40). Cavitation, if present, could enhance focal heating or contribute to mechanical tissue disruption. Because peak positive and negative pressures, acoustic emissions, and cavitation activity were not measured, mechanical disruption and cavitation-mediated contributions could neither be confirmed nor excluded in the present study. Following the initial localized injury of the rat model, the longitudinal histology showed a progressive reduction in TIZ depth and redistribution of pigment-containing debris toward the dermo-epidermal junction and crust. Collectively, these observations are consistent with a proposed crust-associated transepidermal elimination route, in which thermally altered tissue and associated exogenous particles are displaced toward the surface during re-epithelialization.

The rat morphometric data demonstrated a time-course process of tissue remodeling. The transient increase in TIZ length at Day 7 aligns with early reactive expansion during wound healing, whereas the progressive reduction in TIZ depth from 1 h to Day 21 indicates progressive contraction and superficialization of the histologically definable injury zone. Because these measurements were obtained from 2D histological sections, the reduction in apparent depth is more plausibly interpreted as a composite consequence of healing and remodeling rather than as direct evidence of simple upward pigment movement alone.

From mechanistic and clinical perspectives, the 20 MHz HIFU approach evaluated here differs from conventional Q-switched and picosecond laser treatment. Laser-based tattoo removal requires selection of an optical wavelength according to the absorption characteristics of the target pigment and induces pigment fragmentation followed by biological clearance. Although Q-switched laser treatment is generally regarded as a relatively safe standard approach, acute complications such as pain, blistering, crusting, and pinpoint hemorrhage, as well as delayed sequelae including pigmentary alteration, paradoxical darkening, allergic reactions, residual pigmentation or ghost images, and occasional scarring or textural change, have been reported (41). In addition, laser tattoo removal can be prolonged and incomplete, particularly for professional tattoos (41). In contrast to conventional Q-switched and picosecond laser platforms, the 20 MHz HIFU paradigm evaluated here is based on localized acoustic energy deposition and focal coagulative injury rather than selective optical absorption by a specific pigment (15, 16, 24–27, 29). The longitudinal rat histology in the present study is consistent with a proposed wound-healing–linked pathway in which thermally altered dermal tissue and associated pigment debris may be incorporated into an epidermal crust and eliminated during re-epithelialization. However, this was interpreted as histology-supported rather than definitive, because the present study did not directly quantify temperature-time histories, *in vivo* acoustic field distributions, macrophage trafficking, keratinocyte-associated extrusion, or lymphatic pigment clearance. Importantly, the long-term reduction in visible contrast observed across black, red, and blue tattoos is consistent with the possibility that HIFU may function as a wavelength-independent, color-agnostic tattoo-clearance strategy.

Previous investigations of 20 MHz HIFU have separately characterized focal lesion formation in skin-equivalent models, local tolerance in minipigs, or exploratory clinical tattoo-treatment outcomes (25–27). The principal contribution of the present study is therefore not a demonstrated improvement in device performance, but an integrated preclinical framework linking energy-dependent *ex vivo* TIZ morphometry, longitudinal rat histopathology, exploratory multispectral 3D follow-up in a Yucatan minipig, and a preliminary wound-care assessment. This stepwise framework enabled physical lesion formation from 20 MHz HIFU to be considered alongside tissue remodeling, pigment redistribution, and surface recovery. The current prototype of the handheld HIFU device did not include integrated optical targeting, direct in-tissue acoustic-field monitoring, or real-time thermometry. Treatment placement therefore remained dependent on the provisional external grid, coupling-layer thickness, contact pressure, skin curvature, and operator-controlled spot positioning. These factors may contribute to variation in focal placement and to incomplete or overlapping treatment coverage. The exposure regimen also differed from previously reported 20 MHz HIFU tattoo protocols. One prior exploratory clinical protocol used 150 ms exposures of 0.4–1.2 J per shot (25), whereas the present feasibility study used a 1,000 ms exposure at 6 W, corresponding to 6 J per shot, with 10 repeated exposures at each treatment site. The longer exposure duration and repeated-site dosing may promote sustained coagulative injury and the proposed crust-associated pigment-extrusion process, but may also increase cumulative tissue heating, thermal diffusion, and overlap of adjacent thermally altered regions (26, 29).

A clinically relevant limitation emerging from the rat model is that complete short-term surface normalization was not universal after a single 60 J session. The ring-shaped yellow–brown residual traces at Day 21 are possibly compatible with post-inflammatory color change and/or localized peripheral remodeling around the treated spot. These phenomena align with the broader spectrum of delayed cosmetic normalization commonly described after dermatological tattoo interventions (41). This highlights a likely dose–response trade-off, in which higher single-session energy may improve visible pigment reduction but at the cost of delayed surface recovery. This consideration directly motivated the minipig study, which was used to characterize surface-weighted healing trajectories and to explore whether wound-care modulation could improve the early post-treatment course.

The acute minipig trajectories showed divergent early optical and volumetric profiles between normal and tattooed skin. These differences raise the possibility that exogenous pigment may influence both local energy deposition and the surface optical/topographic readouts captured by multispectral imaging. One possible explanation is that pigment-associated alteration of acoustic propagation may favor more superficial energy dissipation, thereby contributing to earlier plateauing of the volumetric response in tattooed skin. In addition, the apparent rapid reduction in redness may partly reflect optical masking of underlying dermal vasodilation by dense dark pigment and superficial whitening in the Antera3D readout.

In the chronic minipig comparison, both normal and tattooed sites showed large early deviations in pigmentation- and redness-related readouts at Day 4, consistent with the acute post-treatment inflammatory phase. Thereafter, recovery trajectories diverged, with the tattooed site retaining a persistent central brownish residual feature and relatively higher localized pigmentation values between Days 25 and 73, suggesting delayed local surface normalization relative to normal skin. Although the present data do not permit direct confirmation of the underlying mechanism, this focal residual pattern may be consistent with heterogeneous local thermal remodeling in the presence of retained tattoo particles, and may also reflect localized post-inflammatory hyperpigmentation. By Day 98, however, both pigmentation- and redness-related metrics had generally returned toward baseline, and surface-height changes remained within a sub-millimeter range. Taken together, these findings confirm a safe recovery course without gross hypertrophic surface distortion or permanent pigmentary sequelae under the studied conditions.

Importantly, long-term contrast reduction and recovery of lightness were observed across red, black, and blue tattoos, supporting the feasibility of a clearance strategy that is not restricted to a single pigment color. This observation is consistent with the conceptual advantage of HIFU over wavelength-dependent laser platforms, because the treatment mechanism does not rely on optical matching between a selected laser wavelength and a specific pigment.

The exploratory dressing comparison further suggests that early wound care may influence the short-term recovery trajectory after high-energy HIFU exposure. Hydrocolloid dressings are described as maintaining a moist wound environment that can support autolytic debridement and re-epithelialization (42). Moreover, a recent randomized clinical trial in dermatologic surgery demonstrated that compared to standard petroleum ointment, hydrocolloid dressings significantly accelerated the resolution of postoperative erythema and improved long-term scar profiles (43). Similarly, in our feasibility test with the hydrocolloid-covered condition, the treated site showed earlier movement toward baseline color and height parameters, along with reduced redness by Day 14, whereas the uncovered site remained darker, redder, and higher over the same interval. These findings suggest that moist occlusive care may accelerate eschar turnover and improve the early recovery course, although this interpretation remains preliminary because of the limited sample size and the sensitivity of optical measurements to ROI definition and contact geometry.

Several limitations should be acknowledged. This study did not include a direct comparator arm using conventional Q-switched or picosecond laser treatment. Consequently, the present study cannot determine whether 20 MHz HIFU provides comparable or superior pigment reduction, fewer treatment sessions, faster recovery, improved cosmetic outcomes, or a more favorable adverse-event profile relative to established laser-based approaches. The rat and minipig experiments were feasibility-oriented, and the minipig component was limited to a single animal. Therefore, the minipig data cannot support statistically reliable conclusions regarding efficacy, reproducibility, safety margins, or generalizability. Furthermore, only red, black, and blue commercial pigments were evaluated, and their detailed chemical compositions and particulate characteristics were not independently characterized. This component instead was interpreted as an exploratory longitudinal imaging dataset describing within-subject surface recovery and pigment-contrast trajectories after 20 MHz HIFU exposure. The absence of formal between-animal replication, randomized site allocation, sham/vehicle-treated controls, and independently powered endpoint analysis is a major limitation. In addition, the minipig imaging outcomes are surface-weighted and algorithm-dependent, with additional uncertainty when exogenous pigments introduce spectral overlap or optical masking. The study did not directly map the in-tissue acoustic field or temperature-time history during exposure, nor did it quantify cellular clearance pathways such as macrophage trafficking, keratinocyte-associated extrusion, or lymphatic transport. This limitation is particularly important because high-frequency ultrasound propagation in skin may be affected by coupling thickness, local contact pressure, tissue curvature, epidermal and dermal acoustic heterogeneity, perfusion-mediated heat removal, and wound-related changes in tissue hydration and collagen density (30, 31, 38, 39). In addition, optical readouts in tattooed skin may be influenced by spectral overlap or masking from exogenous pigments. Accordingly, the proposed ablation-extrusion pathway was interpreted as a histology-supported working model rather than a definitive mechanistic demonstration. Our future studies, therefore, will include adequately powered large-animal cohorts, sham/untreated controls, randomized site allocation, prespecified blinded endpoint analysis, and combine acoustic field mapping, real-time thermal monitoring, and immunohistochemical assays for inflammatory and phagocytic cell populations to establish the causal sequence linking focal thermal injury, pigment redistribution, crust formation, and clearance (29). Finally, the 60 J single-session design was selected to demonstrate feasibility rather than to represent an optimized clinical treatment strategy. Future work should therefore address dose fractionation, spot spacing, wound-care optimization, longer follow-up, and larger cohort studies to better balance clearance efficacy against residual discoloration and remodeling-related sequelae.

## 5 Conclusion

In this feasibility study, 20 MHz HIFU produced localized thermal injury *ex vivo*, with energy-dependent expansion of the TIZ and substrate-dependent differences in axial extent. *In vivo*, macroscopic and histopathological time courses in the rat model were consistent with a proposed crust-associated transepidermal elimination pathway for multicolor dermal pigments after a single 60 J exposure. However, peripheral residual traces indicated that macroscopic normalization may be incomplete within a 21-day window at this dose. In the single-minipig exploratory model, multispectral 3D imaging provided descriptive surface-weighted trajectories of acute vascular and topographical responses followed by gradual long-term recovery. These within-subject observations were accompanied by reduced visible color contrast across multiple tattoo colors at >100 days, but were interpreted as preliminary evidence of feasibility rather than definitive evidence of efficacy. Furthermore, short-term hydrocolloid dressing coverage was associated with earlier eschar detachment and faster normalization of surface color and topography in a preliminary wound-care comparison. In future studies, we will conduct larger controlled cohorts, protocol optimization (including dose titration, coverage area, and fractionation), and extend follow-up with orthogonal outcome measures to determine the reproducibility, safety margins, and clinical applicability of this candidate non-optical approach to reducing tattoo pigments without pigment-specific optical wavelength matching.

## Data Availability

The original contributions presented in the study are included in the article. Further inquiries can be directed to the corresponding authors.

## References

[1] Citrin DE. Recent developments in radiotherapy. N Engl J Med. (2017) 377(11):1065–75. 10.1056/NEJMra1608986 28902591

[2] Bregnhøj A Hædersdal M. Q-switched YAG laser vs. punch biopsy excision for iatrogenic radiation tattoo markers—a randomized controlled trial. J Eur Acad Dermatol Venereol. (2010) 24(10):1183–6. 10.1111/j.1468-3083.2010.03617.x 20236380

[3] Rossi AM Hibler BP Navarrete-Dechent C Lacouture ME. Restorative oncodermatology: diagnosis and management of dermatologic sequelae from cancer therapies. J Am Acad Dermatol. (2021) 85(3):693–707. 10.1016/j.jaad.2020.08.005 32781177 PMC7868476

[4] Hardy-Abeloos C Gorovets D Lewis A Ji W Lozano A Tung CC. Prospective evaluation of patient-reported outcomes of invisible ink tattoos for the delivery of external beam radiation therapy: the PREFER trial. Front Oncol. (2024) 14:1374258. 10.3389/fonc.2024.1374258 38590650 PMC10999588

[5] Runowicz CD Leach CR Henry NL Henry KS Mackey HT Cowens-Alvarado RL. American cancer society/American society of clinical oncology breast cancer survivorship care guideline. CA-Cancer J Clin. (2016) 66(1):43–73. 10.3322/caac.21319 26641959

[6] Kinahan KE Sharp LK Seidel K Leisenring W Didwania A Lacouture ME. Scarring, disfigurement, and quality of life in long-term survivors of childhood cancer: a report from the childhood cancer survivor study. J Clin Oncol. (2012) 30(20):2466–74. 10.1200/JCO.2011.39.3611 22614987 PMC3397782

[7] Lastrucci A Marrazzo L Meattini I Simontacchi G Wandael Y Talamonti C. Advancing patient setup: a comprehensive scoping review of tattoo-less techniques in radiation therapy. Crit Rev Oncol/Hematol. (2024) 204:104518. 10.1016/j.critrevonc.2024.104518 39299409

[8] Svestad JG Heydari M Mikalsen SG Flote VG Nordby F Hellebust TP. Surface-guided positioning eliminates the need for skin markers in radiotherapy of right sided breast cancer: a single center randomized crossover trial. Radiother Oncol. (2022) 177:46–52. 10.1016/j.radonc.2022.10.017 36309152

[9] Li G. Advances and potentials of optical surface imaging in radiotherapy. Phys Med Biol. (2022) 67(16):16TR02. 10.1088/1361-6560/ac838f PMC10958463 35868290

[10] Hoisak JDP Pawlicki T. The role of optical surface imaging systems in radiation therapy. Semin Radiat Oncol. (2018) 28(3):185–93. 10.1016/j.semradonc.2018.02.003 29933878

[11] Zawada T Bove T. Strongly focused HIFU transducers with simultaneous optical observation for treatment of skin at 20 MHz. Ultrasound Med Biol. (2022) 48(7):1309–27. 10.1016/j.ultrasmedbio.2022.03.002 35410743

[12] Wurstbauer K Sedlmayer F Kogelnik HD. Skin markings in external radiotherapy by temporary tattooing with henna: improvement of accuracy and increased patient comfort. Int J Radiat Oncol Biol Phys. (2001) 50(1):179–81. 10.1016/S0360-3016(01)01439-0 11316562

[13] Goto M Oshiro Y Tamaki Y Ishida T Kato Y Shinoda K. A novel method for skin marking in radiotherapy: first clinical use of temporary organic tattoo seal. J Radiat Res. (2022) 63(2):314–8. 10.1093/jrr/rrab126 35067716 PMC8944313

[14] Landeg SJ Kirby AM Lee SF Bartlett F Titmarsh K Donovan E. A randomized control trial evaluating fluorescent ink versus dark ink tattoos for breast radiotherapy. Br J Radiol. (2016) 89(1068):20160288. 10.1259/bjr.20160288 27710100 PMC5604906

[15] Gurnani P Williams N Al-Hetheli G Chukwuma O Roth R Fajardo F. Comparing the efficacy and safety of laser treatments in tattoo removal: a systematic review. J Am Acad Dermatol. (2022) 87(1):103–9. 10.1016/j.jaad.2020.07.117 32763326

[16] Kassirer S Zachary CB Marini L Adatto M Landau M. Laser tattoo removal strategies: part II: a review of the methods, techniques, and complications involved in tattoo removal. J Am Acad Dermatol. (2025) 93(1):19–32. 10.1016/j.jaad.2024.05.097 38980249

[17] Bäumler W Weiß KT. Laser assisted tattoo removal—state of the art and new developments. Photochem Photobiol Sci. (2019) 18(2):349–58. 10.1039/c8pp00416a 30452057

[18] Choi MS Seo HS Kim JG Choe SJ Park BC Kim MH. Effects of picosecond laser on the multi-colored tattoo removal using Hartley Guinea pig: a preliminary study. PLoS One. (2018) 13(9):e0203370. 10.1371/journal.pone.0203370 30188934 PMC6126847

[19] Cecchetti D Bauer EM Guerriero E Sennato S Tagliatesta P Tagliaferri M. Comparative treatments of a green tattoo ink with Ruby, Nd:YAG nano- and picosecond lasers in normal and array mode. Sci Rep. (2022) 12(1):3571. 10.1038/s41598-022-07021-w 35246552 PMC8897463

[20] Choudhary S Elsaie ML Leiva A Nouri K. Lasers for tattoo removal: a review. Lasers Med Sci. (2010) 25(5):619–27. 10.1007/s10103-010-0800-2 20549279

[21] Kurniadi I Tabri F Madjid A Anwar AI Widita W. Laser tattoo removal: fundamental principles and practical approach. Dermatol Ther. (2021) 34(1):e14418. 10.1111/dth.14418 33068020

[22] Bäumler W Breu C Philipp B Haslböck B Berneburg M Weiß KT. The efficacy and the adverse reactions of laser-assisted tattoo removal—a prospective split study using nanosecond and picosecond lasers. J Eur Acad Dermatol Venereol. (2022) 36(2):305–12. 10.1111/jdv.17674 34543473

[23] Hernandez L Mohsin N Frech FS Dreyfuss I Vander Does A Nouri K. Laser tattoo removal: laser principles and an updated guide for clinicians. Lasers Med Sci. (2022) 37(6):2581–7. 10.1007/s10103-022-03576-2 35604505

[24] Laux P Tralau T Tentschert J Blume A Dahouk SA Bäumler W. A medical-toxicological view of tattooing. Lancet. (2016) 387(10016):395–402. 10.1016/S0140-6736(15)60215-X 26211826

[25] Serup J Bove T Zawada T Jessen A Poli M. High-frequency (20 MHz) high-intensity focused ultrasound: new ablative method for color-independent tattoo removal in 1–3 sessions. An open-label exploratory study. Skin Res Technol. (2020) 26(6):839–50. 10.1111/srt.12885 32557859 PMC7754334

[26] Bove T Zawada T Serup J Jessen A Poli M. High-frequency (20 MHz) high-intensity focused ultrasound (HIFU) system for dermal intervention: preclinical evaluation in skin equivalents. Skin Res Technol. (2019) 25(2):217–28. 10.1111/srt.12661 30620418

[27] Soegaard S Aarup V Serup J Bove T Zawada T Jessen A. High-frequency (20 MHz) high-intensity focused ultrasound system for dermal intervention: a 12-week local tolerance study in minipigs. Skin Res Technol. (2020) 26(2):241–54. 10.1111/srt.12786 31541524

[28] Woźniak B Sobolewski P Sauer N Koper M Calik J. High-intensity focused ultrasound in dermatology: a review with emphasis on skin cancer management and prevention. Cancers (Basel). (2025) 17(21):3518. 10.3390/cancers17213518 PMC12610741 41228308

[29] Rivens I Jayadewa C Mouratidis P Ter Haar G. Histological characterization of HIFU lesions. Int J Hyperthermia. (2024) 41(1):2389292. 10.1080/02656736.2024.2389292 39134063

[30] Moran CM Bush NL Bamber JC. Ultrasonic propagation properties of excised human skin. Ultrasound Med Biol. (1995) 21(9):1177–90. 10.1016/0301-5629(95)00049-6 8849832

[31] El Gammal S El Gammal C Kaspar K Pieck C Altmeyer P Vogt M. Sonography of the skin at 100 MHz enables *in vivo* visualization of stratum corneum and viable epidermis in palmar skin and psoriatic plaques. J Invest Dermatol. (1999) 113(5):821–9. 10.1046/j.1523-1747.1999.00795.x 10571740

[32] Kim H-J Kim HG Zheng Z Park HJ Yoon JH Oh W. Coagulation and ablation patterns of high-intensity focused ultrasound on a tissue-mimicking phantom and cadaveric skin. Lasers Med Sci. (2015) 30(9):2251–8. 10.1007/s10103-015-1804-8 26341380

[33] Matias AR Ferreira M Costa P Neto P. Skin colour, skin redness and melanin biometric measurements: comparison study between Antera 3D®, Mexameter® and Colorimeter®. Skin Res Technol. (2015) 21(3):346–62. 10.1111/srt.12199 25645051

[34] Linming F Wei H Anqi L Yuanyu C Heng X Sushmita P. Comparison of two skin imaging analysis instruments: the VISIA® from Canfield vs the ANTERA 3D®CS from Miravex. Skin Res Technol. (2018) 24(1):3–8. 10.1111/srt.12381 28585335

[35] Liu H Yang J Huang X Yi Z Rao Y Li J. Antera 3D® skin image analysis system for objective evaluation of clinical treatment efficacy in a patient with facial vitiligo. Arch Dermatol Res. (2025) 317(1):594. 10.1007/s00403-025-03941-5 40100282

[36] Smith SW Trahey GE von Ramm OT. Phased array ultrasound imaging through planar tissue layers. Ultrasound Med Biol. (1986) 12(3):229–43. 10.1016/0301-5629(86)90314-5 3962008

[37] Finlayson L Barnard IRM McMillan L Ibbotson SH Brown CTA Eadie E. Depth penetration of light into skin as a function of wavelength from 200 to 1000 nm. Photochem Photobiol. (2022) 98(4):974–81. 10.1111/php.13550 34699624

[38] Olerud JE O'Brien W Riederer-Henderson MA Steiger D Forster FK Daly C. Ultrasonic assessment of skin and wounds with the scanning laser acoustic microscope. J Invest Dermatol. (1987) 88(5):615–23. 10.1111/1523-1747.ep12470221 3553343

[39] Olerud JE O'Brien WD Jr Riederer-Henderson MA Steiger DL Debel JR Odland GF. Correlation of tissue constituents with the acoustic properties of skin and wound. Ultrasound Med Biol. (1990) 16(1):55–64. 10.1016/0301-5629(90)90086-R 2321315

[40] Ter Haar G Coussios C. High intensity focused ultrasound: physical principles and devices. Int J Hyperthermia. (2007) 23(2):89–104. 10.1080/02656730601186138 17578335

[41] Khunger N Molpariya A Khunger A. Complications of tattoos and tattoo removal: stop and think before you ink. J Cutan Aesthet Surg. (2015) 8(1):30–6. 10.4103/0974-2077.155072 25949020 PMC4411590

[42] Field CK Kerstein MD. Overview of wound healing in a moist environment. Am J Surg. (1994) 167(1A):S2–6. 10.1016/0002-9610(94)90002-7 8109679

[43] Bell MC Gangodawila TW Morr CS Xue GR Iqbal A Merkel EA. Hydrocolloid dressing vs petroleum ointment for scar appearance after dermatologic surgery: a randomized clinical trial. JAMA Dermatol. (2025) 161(12):1246–51. 10.1001/jamadermatol.2025.4051 41123900 PMC12547672

